# Understanding of bacterial lignin extracellular degradation mechanisms by *Pseudomonas putida* KT2440 via secretomic analysis

**DOI:** 10.1186/s13068-022-02214-x

**Published:** 2022-10-31

**Authors:** Zhangyang Xu, Bo Peng, Reta Birhanu Kitata, Carrie D. Nicora, Karl K. Weitz, Yunqiao Pu, Tujin Shi, John R. Cort, Arthur J. Ragauskas, Bin Yang

**Affiliations:** 1 Bioproducts, Sciences & Engineering Laboratory, Department of Biological Systems Engineering, ashington State University Tri-Cities, Joint Appointment: Pacific Northwest National Laboratory, 2710 Crimson Way, Richland, WA 99354 USA; 2 Biological Sciences Division, Pacific Northwest National Laboratory, Richland, Washington 99352 USA; 3 Joint Institute for Biological Sciences, Biosciences Division, Oak Ridge National Laboratory, Oak Ridge, TN 37831 USA; 4 Department of Chemical and Biomolecular Engineering, University of Tennessee, Knoxville, TN 37996 USA; 5 Department of Forestry, Wildlife, and Fisheries, Center for Renewable Carbon, University of Tennessee Institute of Agriculture, Knoxville, TN 37996 USA

**Keywords:** Lignin degradation, *Pseudomonas putida*, Secretome, NMR, Proteomics

## Abstract

**Background:**

Bacterial lignin degradation is believed to be primarily achieved by a secreted enzyme system. Effects of such extracellular enzyme systems on lignin structural changes and degradation pathways are still not clearly understood, which remains as a bottleneck in the bacterial lignin bioconversion process.

**Results:**

This study investigated lignin degradation using an isolated secretome secreted by *Pseudomonas putida* KT2440 that grew on glucose as the only carbon source. Enzyme assays revealed that the secretome harbored oxidase and peroxidase/Mn^2+^-peroxidase capacity and reached the highest activity at 120 h of the fermentation time. The degradation rate of alkali lignin was found to be only 8.1% by oxidases, but increased to 14.5% with the activation of peroxidase/Mn^2+^-peroxidase. Gas chromatography–mass spectrometry (GC–MS) and two-dimensional ^1^H–^13^C heteronuclear single-quantum coherence (HSQC) NMR analysis revealed that the oxidases exhibited strong C–C bond (*β-β*, *β*-5, and *β*-1) cleavage. The activation of peroxidases enhanced lignin degradation by stimulating C–O bond (*β*-O-4) cleavage, resulting in increased yields of aromatic monomers and dimers. Further mass spectrometry-based quantitative proteomics measurements comprehensively identified different groups of enzymes particularly oxidoreductases in *P. putida* secretome, including reductases, peroxidases, monooxygenases, dioxygenases, oxidases, and dehydrogenases, potentially contributed to the lignin degradation process.

**Conclusions:**

Overall, we discovered that bacterial extracellular degradation of alkali lignin to vanillin, vanillic acid, and other lignin-derived aromatics involved a series of oxidative cleavage, catalyzed by active DyP-type peroxidase, multicopper oxidase, and other accessory enzymes. These results will guide further metabolic engineering design to improve the efficiency of lignin bioconversion.

**Graphical Abstract:**

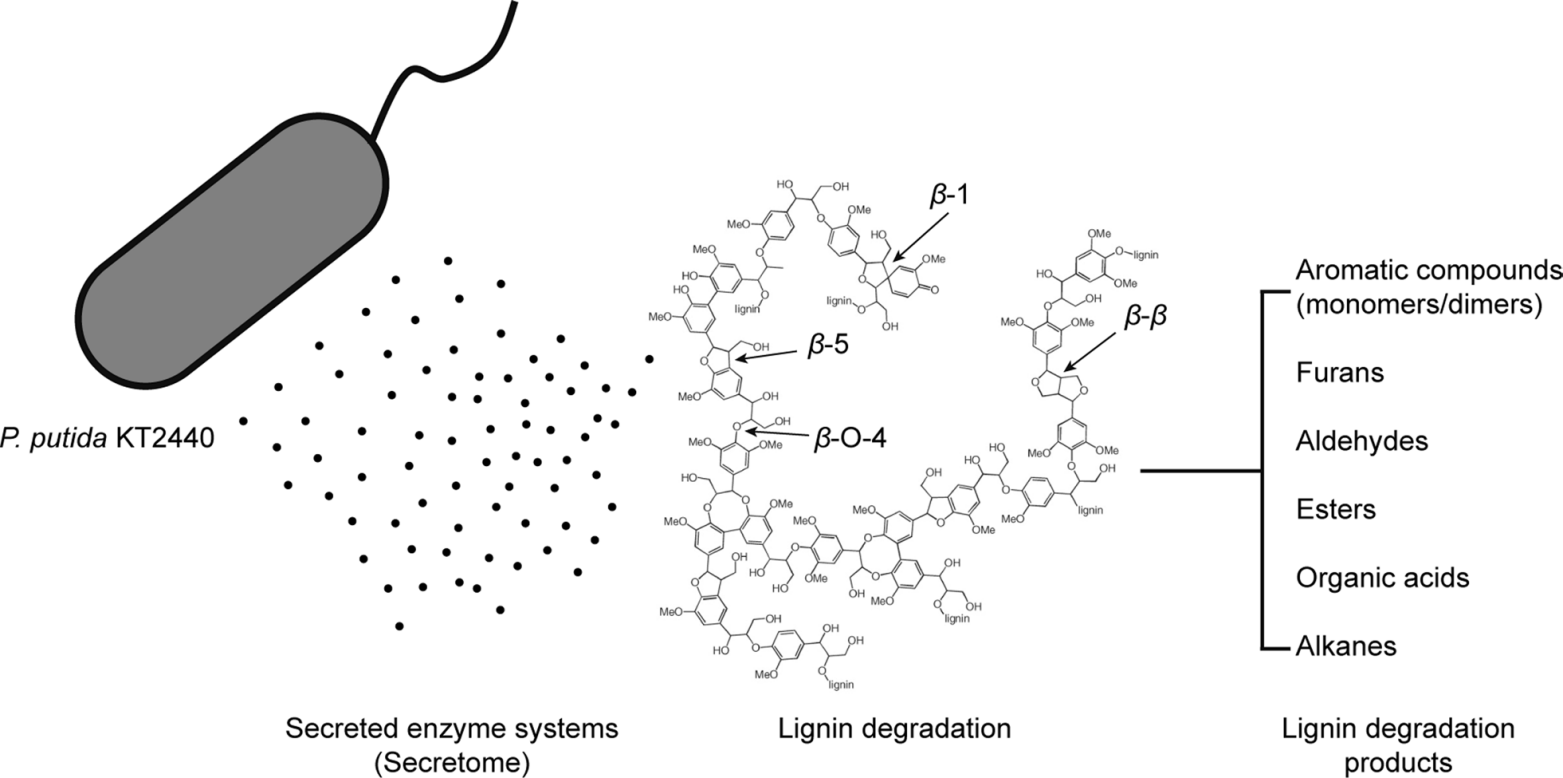

**Supplementary Information:**

The online version contains supplementary material available at 10.1186/s13068-022-02214-x.

## Background

The current vision of biorefinery undervalues lignin’s potential to address this nation’s high demands on quality liquid fuel, chemicals and biomaterials [1–8]. Lignin is an abundant natural component from plant biomass, but remains challenging in bioconversion process due to its recalcitrant polymeric structure [9, 10], while the highly heterogeneous linkages among its aromatic centers, including a variety of C–O–C bonds (*β*-O-4/4′, *α*-O-4/4′, *α/γ*-O-*γ*, 4-O-5/5′, etc.) and C–C bonds (5–5/5′, *β-β*, *β*-1, *β*-5, etc.) [1–3]. To improve the bioconversion of lignocellulosic feedstocks, more effective lignin degradation methods are in demand. Approaches published for lignin degradation and conversion include chemical degradation and enzymatic lignin breakdown [4, 11–22]. Compared to the chemical and thermal degradation, enzymatic lignin breakdown is low cost and environmentally friendly [4, 5, 23]. Nature has found ways to degrade lignin by producing dedicated ligninolytic enzyme systems [24, 25]. While such enzymes have been thoroughly studied for ligninolytic fungi [26–28], reports on bacterial enzymes capable of lignin modification have been relatively new in recent years [13, 27, 29, 30]. Several types of oxidoreductases that enable bacteria to act on lignin have been revealed [12, 31–34].

As a lignin-degrading bacteria, *Pseudomonas putida* KT2440 has catabolic potential against a wide range of natural aromatic compounds. Linger et al. demonstrated the lignin breakdown products bioconversion capacity in *P. putida* KT2440 [35]. The presence of *P. putida* KT2440 with fungal secretome enhanced lignin degradation possible due to the catabolism of low-molecular-weight lignin and partially prevent of repolymerization [36]. Continuous oxidative enzymes that might be involved in lignin break down processes are identified consistently in *P. putida*. Screening studies reported *P. putida* KT2440 secreted ligninolytic enzymes such as laccase, Mn^2+^-independent peroxidase (e.g., DyP), and Mn^2+^-oxidizing peroxidase (e.g., MnP and DyP) [17]. Multicopper oxidase (CopA) from *P. putida* KT2440 was characterized afterward with the lignin model compounds oxidizing ability [37].Furthermore, *β*-etherase (glutathione S-transferases, GSTs) and dioxygenases (e.g., 2,3-quercetin dioxygenase) were upregulated in the secretome of *P. putida* when exposed to lignin. Moreover, multicopper oxidase such as CopA was also detected in the secretome when *P. putida* grew on lignin-free media (glucose), suggesting the possibility of other carbon sources (e.g., glucose) could also induce oxidoreductases that might be involved in lignin catabolism process. In addition, spatiotemporal mechanisms for lignin catabolism in *P. putida* KT2440 have revealed that outer membrane vesicle (OMVs) would encapsulate enzymes involved in the catabolism of lignin-derived aromatic structures [33]. However, the oxidative lignin degradation enzymes in *P. putida* KT2440 are under-identified, which requires more efforts to explore potential enzymes.

Understanding the extracellular degradation pathways and lignin structural changes by *Pseudomonas putida* KT2440 is critical for developing processes for efficient lignin bioconversion. Lignin structural changes have been investigated in the presence of *P. putida*. For example, ^13^P NMR results revealed the *β*-5 phenolic group from lignin significantly decreased when Dyp was overexpressed in *P. putida* [38]. 2D NMR results revealed ferulic acid and p-coumaric acid signals from lignin disappeared at 72 h of fermentation in *P. putida* KT2440 [33]. Although bacterial lignin conversion examples are accumulating, most of the studies fermented the wild-type or mutated *P. putida* together with lignin which overlapped the function of secreted enzyme system with the strain. The specific function of extracellular enzyme systems on lignin structural change and degradation pathway is still unknown in *P. putida* compared to fungi cocktails [33, 36, 39].

In this study, we tracked the activity of oxidases and peroxidase/Mn^2+^-peroxidase in the secretome of *Pseudomonas putida* KT2440 that grew on glucose as the only carbon source. The active secretome was isolated to react with alkali lignin in order to measure the lignin degradation rate with or without the addition of H_2_O_2_ and Mn^2+^. The degraded lignin products and structural change were analyzed by GC–MS and NMR to investigate the possible reaction mechanisms of lignin degradation by secretome in *P. putida* KT2440. In addition, global proteomics analysis was carried out for secretome and intracellular proteome to identify potential enzymes involved in the lignin degradation process (Additional file 1: Fig. S1).

## Results

### Cell growth and concentration of extracellular and intracellular protein

To generate an effective secretome with active extracellular enzymes in *Pseudomonas putida*, *P. putida* cells were first grown on M9 medium with 5 g·L^−1^ glucose and 1 g·L^−1^ NH_4_Cl for 8 days. The optical density (OD600) and extracellular and total protein (intracellular + extracellular) concentrations were measured.

**Fig. 1:**
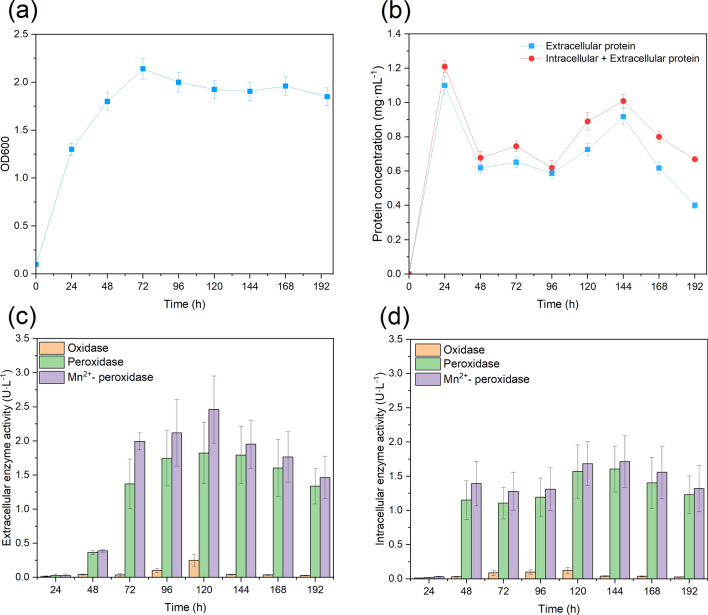
Cell growth (**a**) and concentrations of extracellular and total protein (intracellular + extracellular protein) (**b**). Profiles of extracellular (**c**) and intracellular (**d**) ligninolytic enzymes in *P. putida* over time

Figure 1 depicts the cell density (OD600), and extracellular and total protein concentrations over time. As shown in Fig. 1a, with 5 g·L^−1^ glucose, cells grew fast for the first 72 h of fermentation, and the optical density reached 2.1 and maintained a similar level until the end of the fermentation.

A distinct pattern was revealed for protein concentration. As shown in Fig. 1b (blue line), the extracellular protein concentration reached 1.1 mg·mL^−1^ at 24 h and dropped to 0.6 mg·mL^−1^ at 48 h, then gradually increased to 0.9 mg·mL^−1^ at 144 h and further decreased to 0.4 mg·mL^−1^ at the end of fermentation. The same trend was also observed for total protein concentration (red line). The total protein concentration reached 1.2 mg·mL^−1^ at 24 h and dropped to 0.7 mg·mL^−1^ at 48 h, then gradually increased to 1.0 mg·mL^−1^ at 144 h and further decreased to 0.7 mg·mL^−1^ at the end of fermentation. The total protein concentrations were higher than the extracellular protein, including the lysate intracellular protein. These results indicated that the *P. putida* cells secreted protein to extracellular space and the extracellular protein concentration fluctuated over time. However, further enzyme activity assays are still required to examine whether these proteins are secreted as active form or not.

### Extracellular vs. intracellular enzyme activity

*P. putida* cells secreted proteins to extracellular space when grown on glucose. However, whether the secretome harbors active ligninolytic enzymes still requires specific investigation. The phenolic substrate 2,6-dimethoxyphenol (DMP) is usually used to track laccase-like oxidases and peroxidase activities [17]. Therefore, oxidases, peroxidases, and Mn^2+^-oxidizing peroxidases were tracked in the culture supernatants and intracellular over 8-day incubations. In Fig. 1c, extracellular oxidase activity was detected at a low level at 24 h, and gradually increased to 0.25 U/L at 120 h. In Fig. 1d, the profiles for intracellular enzymes are similar to extracellular enzymes. The oxidase activity was maintained at a low level at 24 h and then gradually increased to 0.12 U/L at 120 h.

The peroxidase activity (e.g., Dyp) was also measured with DMP and H_2_O_2_ addition [17]. In Fig. 1c, extracellular peroxidase activity was detected at a low level at 24 h and gradually increased to 1.8 U/L at 120 h. In Fig. 1d, the profiles for intracellular enzymes are similar to extracellular enzymes. The peroxidase activity was maintained at a low level at 24 h and then gradually increased to 1.6 U/L at 120 h.

DMP was also used to measure the reaction’s Mn^2+^-peroxidase activity with H_2_O_2_ and Mn^2+^ [17]. In Fig. 1c, extracellular peroxidase activity was detected at a low level at 24 h, and gradually increased to 2.5 U/L at 120 h. In Fig. 1d, the profiles for intracellular enzymes are similar to extracellular enzymes. The Mn^2+^-peroxidase activity was maintained at a low level at 24 h and then gradually increased to 1.7 U/L at 120 h.

Collectively, the results in Fig. 1c demonstrate that *P. putida* secreted the enzyme to the extracellular space, and the maximum enzyme activity appeared at 120 h of fermentation. The intracellular enzyme activity showed a similar pattern as extracellular in Fig. 1d, with the maximum enzyme activity at 120 h. Therefore, the extracellular enzyme at 120 h of fermentation was selected for the following lignin degradation reaction.

### Amount of alkali lignin degraded

**Fig. 2:**
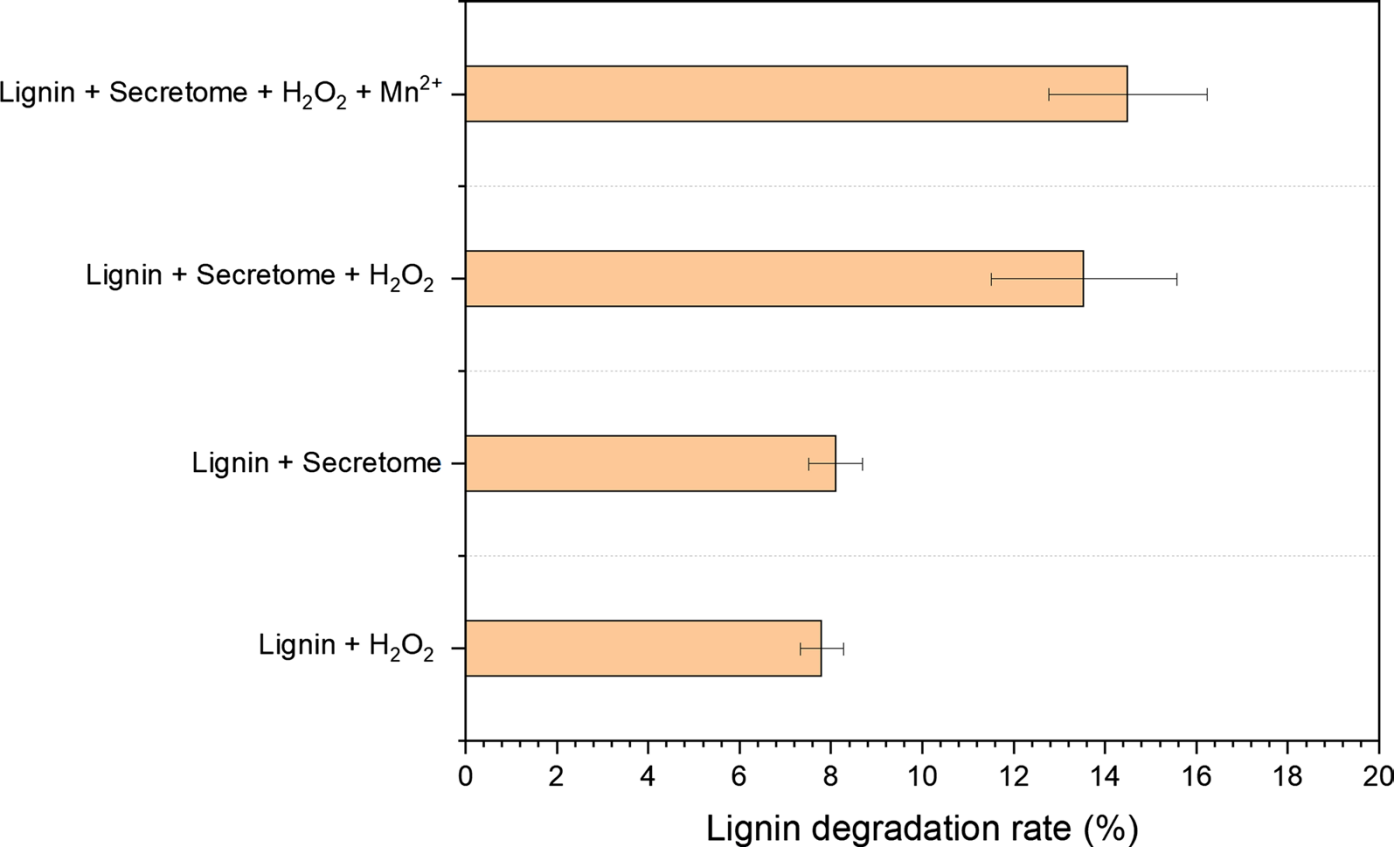
Degradation rate of alkali lignin by secretome alone and in the presence of H_2_O_2_ and Mn^2+^. The degradation rates were measured by 120 h of treatment (n = 3)

The lignin degradation reaction solution was set up with secretome and 2 g·L^−1^ alkali lignin with or without 0.1 mM H_2_O_2_ and 0.1 mM Mn^2+^ addition and reacted for 5 days. Apart from the secretome alone treatment, H_2_O_2_ and/or Mn^2+^ were added separately to activate the capacity of peroxidase and Mn^2+^-peroxidase performance on lignin degradation. In Fig. 2, when individual secretome was used for lignin degradation reaction, the amount of alkali lignin degraded only reached 8.1%, which was substantially lower than that for either H_2_O_2_ or H_2_O_2_ with Mn^2+^ addition. When H_2_O_2_ was added to the reaction, the amount of alkali lignin degraded reached 13.5%. With the presence of both H_2_O_2_ and Mn^2+^, the alkali lignin degradation rate reached a maximum value of 14.5%. On the other side, the effectiveness of H_2_O_2_ alone has also been tested for comparison, with the alkali lignin degradation rate as of 7.8% only.

### GC–MS analysis of lignin breakdown products

GC–MS analysis was performed to identify the lignin breakdown products with secretome and the addition of H_2_O_2_ and Mn^2+^. The NIST library was used to identify and assign the chromatographic peaks. The amount of each aromatic degradation product was determined from the corresponding peak area in the chromatogram.

The GC–MS chromatogram identified aromatic compounds, furan, aldehydes, esters, organic acids, and alkanes among different samples (Additional file 2: Figs. S2–S4, and Table S1, S2). These identifications should be interpreted as unvalidated candidates; some may be incompatible with known or plausible lignin degradation mechanisms.

Peak area for all observed compounds was analyzed and shown in Additional file 1: Fig. S4, which were further separated as four groups after being compared with their alternatives in lignin control (Additional file 1: Table S2). Notably, when lignin was treated with H_2_O_2_, a wide range of aromatic monomers and dimers (No. 1, 4, 5, 6, 12, 14, 16–18, 20–21) were significantly increased. These compounds were only present in limited amounts or even not detected when treated with secretome alone or in the presence of H_2_O_2_ and Mn^2+^, suggesting the non-specific oxidative cleavage (e.g., H_2_O_2_) showed stronger products release ability compared to enzymatic cleavage. Moreover, the peak area of identified compounds among all the secretome treatments showed a similar distribution pattern (compounds No. 3, 7, 8, 9, 10, 13, 14, 25, 30, 31, 33) but with difference abundance, suggesting the increased compounds might be associated with different activated enzymes. For example, phenol, 2-methoxy-4-propyl- and 3-benzofurancarboxylic acid, 2,3-dihydro-2-methoxy-, methyl ester, trans- (compounds 8 and 13) were increased in secretome alone treatment and maintained the similar level or decreased by H_2_O_2_ and Mn^2+^ addition, indicating these products might be associated with oxidases behavior. Similarly, the peak area of phenol, 4-ethyl-2-methoxy- and acetovanillone (compounds 3 and 9) were enhanced by H_2_O_2_, and vanillic acid (compound 11) was enhanced by Mn^2+^ addition, suggesting these compounds might be associated with peroxidase behavior. How lignin structural changes were impacted by these treatments was further explored by NMR analysis.

### 2D HSQC NMR spectra from the hydrolyzed lignin

**Fig. 3.**
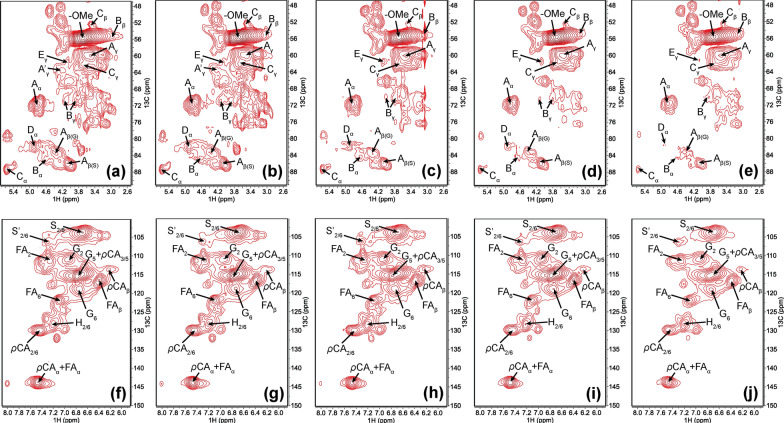
2D ^1^H–^13^C HSQC NMR spectra of lignin with different treatments at 120 h. **a**–**e** Oxygenated aliphatic region; **f**–**j** aromatic region; **a** and **f** lignin control; **b** and **g** lignin with H_2_O_2_; **c** and **h** lignin with secretome; **d** and **i** lignin with secretome and H_2_O_2_; **e** and **j** lignin with secretome and H_2_O_2_ and Mn^2+^. Note: the assignments and NMR-detected lignin linkages are shown in Additional file 1: Table S3 and Fig. S5

The cleavage of lignin C–O–C linkages is vital in the bacterial lignin degradation process. The quantification of each linkage is based on the volume integration of cross-peak contours in the HSQC spectra. In this study, lignin linkages such as *β*-O-4, *β*-*β*, *β*-5, and *β*-1 were cleaved with H_2_O_2_ or secretome treatments (Fig. 3a–j, Table 1 and Additional file 1: Fig. S5, Table S3). As shown in Table 1, in general, the cleavage of *β*-5 was less extensive than the cleavages of *β*-O-4, *β*-*β*, and *β*-1, which indicated that *β*-5 bonds were relatively more stable. The cleavage of these linkages was increased when lignin was treated with H_2_O_2_. Among all the detected linkages, *β*-O-4 bonds exhibited the most extensive cleavage, demonstrating the non-enzymatic chemical reaction with H_2_O_2_ mainly cleavage *β*-O-4 bonds. In contrast, when lignin was treated with secretome alone, *β*-O-4 bonds only exhibited limited cleavage compared to that with H_2_O_2_. While the cleavage of *β*-*β*, *β*-5, and *β*-1 showed increased cleavage compared to that with H_2_O_2_, suggesting the secretome alone may only exhibit oxidase ability and cleavage the C–C bond. When introducing the H_2_O_2_ with the secretome, *β*-O-4 bonds were extensively decreased, *β*-*β* and *β*-1 were further decreased compared to secretome alone. Hydrogen peroxide activates the peroxidase and further cleavages the *β*-O-4 bonds. In addition, the presence of Mn^2+^ further enhanced the peroxidase performance and resulted in further enhanced *β*-O-4, *β*-*β* and *β*-1 bonds cleavage.

**Table 1:** Distribution of the detected linkages of corn stover lignin in different treatments^a^

Inter linkages (%)	*β* aryl ether (*β*-O-4’)	Resinol (*β*-*β*')	Phenylcoumaran (*β*-5')	Spirodienone (*β*-1)
Corn stover lignin control	38.6	3.53	6.81	1.72
Lignin + H_2_O_2_	35.7	3.13	6.56	1.37
Lignin + secretome	37.2	3.16	5.86	1.25
Lignin + secretome + H_2_O_2_	34.2	2.25	5.84	0.97
Lignin + secretome + H_2_O_2_ + Mn^2+^	33.2	1.83	5.70	0.67

^a^Contents were expressed as a percentage relative to the total lignin subunits (G + H + S)

### Secretome and cellular proteome profiles in *P. putida*

**Fig. 4:**
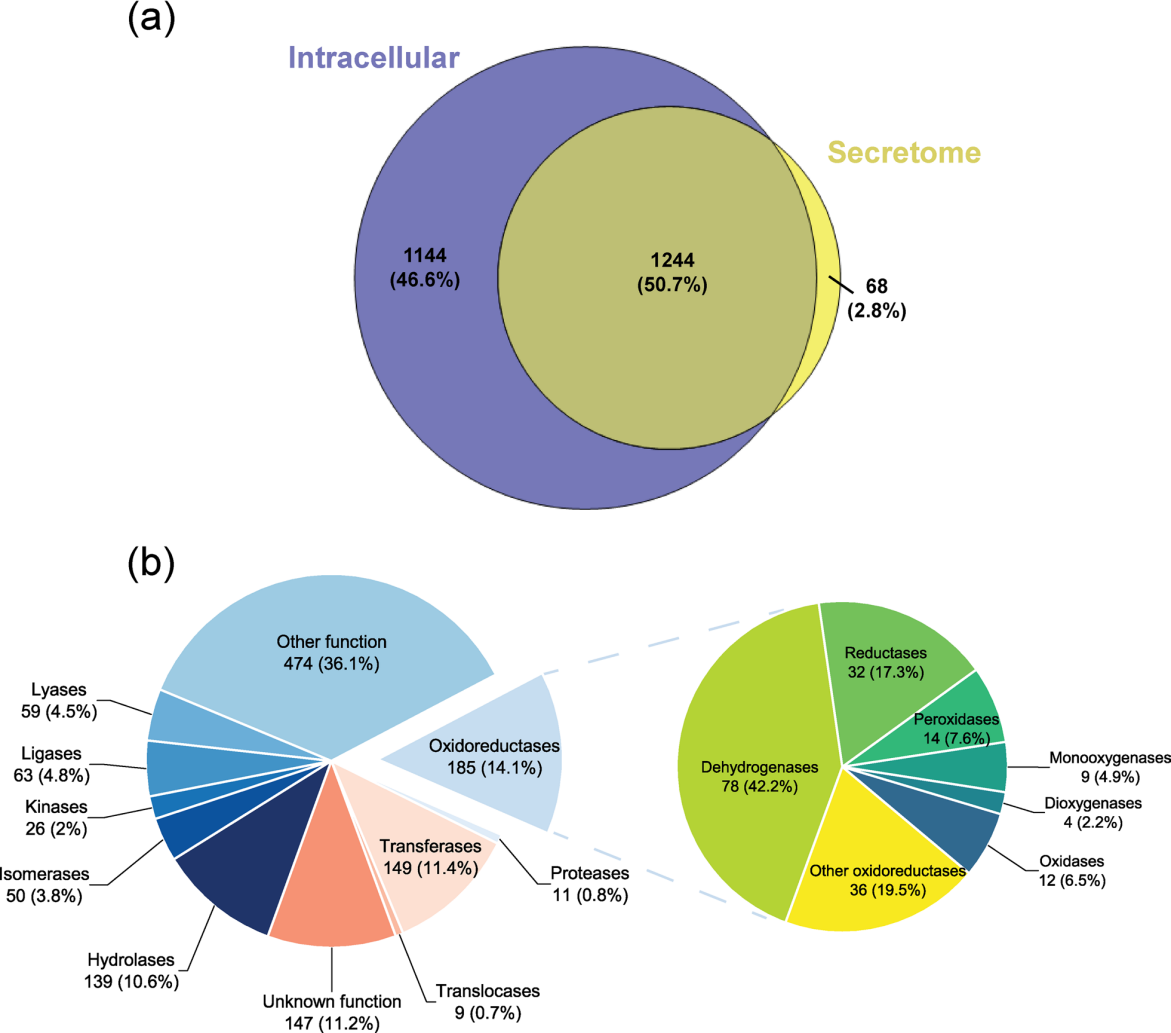
Proteomic analysis of the intracellular and secretome in *P. putida* KT2440. **a** Overlap analysis for intracellular and secretome proteins in *P. putida* KT2440. **b** Functional classification of the proteins detected in the *P. putida* secretome. Classification of the oxidoreductases in the secretome is highlighted in a separate pie chart

GC–MS and NMR results demonstrated that *P. putida* secretome is able to depolymerize lignin. Enzyme assay revealed that the secretome exhibited the oxidase and peroxidase activity. However, isoenzymes with similar activities cannot be distinguished based on simple activity analysis. To deeply characterize the secretome, mass spectrometry-based global proteomics was utilized to profile the proteome of secretome and intracellular extracts in *P. putida* KT2440. Bacterial secretome and cell pellet were harvested at 120 h of fermentation with the highest oxidase and peroxidase activity. Only those proteins presented in more than three replicates were considered as reliable detection and quantitation. Results showed that 1312 proteins were identified in the secretome sample, which was lower than that in the intracellular extracts for 2388 identified proteins. Around 94.8% of the secretome proteins were shared with the intracellular protein, and only 68 proteins were exclusively detected in the secretome (Fig. 4a).

Secretome proteins were then organized by function and presented in Fig. 4b. Despite the other function and unknown function groups, the oxidoreductase group was the most abundant among the rest of the functional groups, which accounted for 14.1% in secretome proteome. Moreover, six glutathione S-transferases were detected and grouped in the transferase group. A deeper analysis of oxidoreductase is presented in Fig. 4b as well. The dehydrogenase group was the most abundant (42.2%), followed by reductase (17.3%), peroxidase (7.6%), oxidase (6.5%), monooxygenase (4.9%), and dioxygenase (2.2%). Notably, there are 14 peroxidases detected, such as Dyp-type peroxidase (PP_3248), cytochrome c551 peroxidase (PP_2943), and alkyl hydroperoxide reductase (e.g., ahpC). Besides, 12 oxidases were detected, including three multicopper oxidases (copA/B and CumA). Besides, a couple of dehydrogenases were detected in the secretome, including NAD(P)H dehydrogenase (e.g., PP_1644), choline dehydrogenase (e.g., betA), aldehyde dehydrogenase (e.g., aldB-I), and alcohol dehydrogenase(e.g., PP_2827). In addition, some hydrogen peroxide alleviating enzymes were also detected, such as catalase (e.g., katG), superoxide dismutase (sodB), and thioredoxin (e.g., trx). Overall, proteomics analysis revealed the oxidoreductase enzymes in the secretome, which not only confirmed the enzyme assay results, but also revealed the specific ability to selectively depolymerize lignin.

### Lignin degradation pathways of *P. putida* secretome

**Fig. 5:**
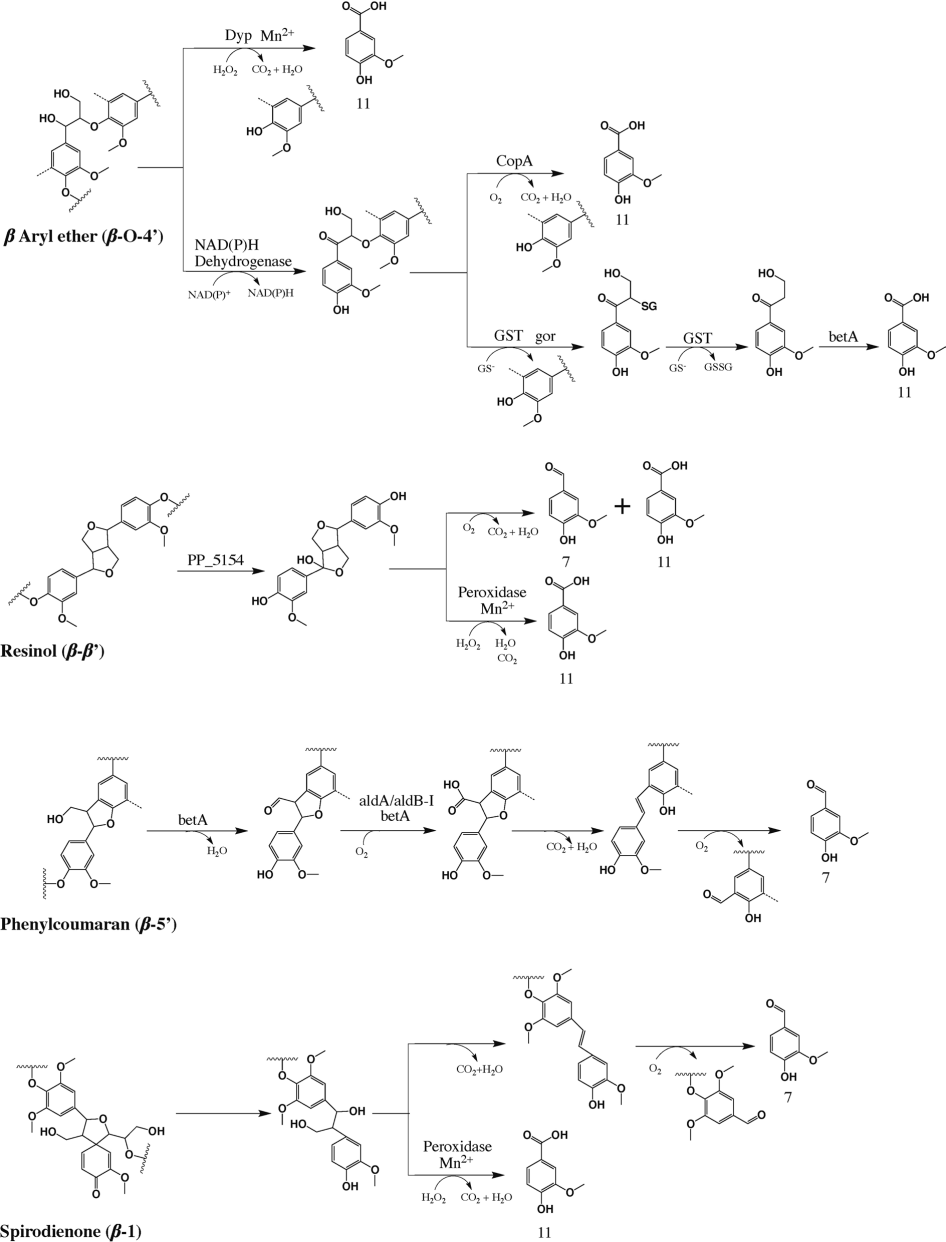
Proposed lignin degradation pathways with *P. putida* secretome. The lignin linkages, final degradation products and enzymes in the pathway map are identified by NMR, GC–MS and proteomics analysis in this study, and the reaction mechanisms are cross-validated by previous literature [27, 37, 40–42, 44, 48, 57, 69]. Abbreviations of enzyme: Dyp, Dyp-type peroxidase (PP_3248); CopA, multicopper oxidase (copA-II); GST, glutathione S-transferase (PP_1644); gor, glutathione reductase; betA, choline dehydrogenase; PP_5154, FAD-binding oxidoreductase; aldA/aldB-I, aldehyde dehydrogenase. Detailed enzyme information can be found in Additional file 2: Table S4

Based on the enzyme activity analysis, GC–MS, NMR, proteomics results, the proposed lignin degradation reaction pathways are shown in Fig. 5 [21, 27, 37, 40–46]. The enzymes identified that might be involved in the lignin catabolism pathway are summarized in Additional file 2: Table S4. Overall, the proposed lignin degradation pathways include cleavage of *β*-aryl ether (*β*-O-4), resinol (*β*-*β*), phenylcoumaran (*β*-5), and spirodienone (*β*-1) linkages. Figure 5 demonstrates that vanillin (compound 7) and vanillic acid (compound 11) are the most important intermediates for all these linkages.

Limited *β*-O-4 bond cleavage (Table 1) was observed when lignin was treated with secretome alone, possibly catalyzed by the NAD(P)H dehydrogenases (similar to LigD/N/L in *Sphingobium sp.* SYK-6) [44] and multicopper oxidase (e.g., CopA) in the enzyme system through *α*-OH oxidation and Cα-Cβ cleavage forming vanillic acid (compound 11). Besides, proteomics results also revealed that glutathione S-transferases (GST), glutathione reductases (gor), and choline dehydrogenase (betA) were presented in the secretome, which functioned as LigE/F, LigG, and HpvZ in *Sphingobium sp.* SYK-6 [42, 44, 47]. Thus, in parallel to the multicopper oxidase route, oxidized *β*-O-4 linkage could also be converted to vanillic acid with the assist from GST, betA, and gor. In addition, NMR results revealed that the presence of H_2_O_2_ with secretome and lignin enhanced the *β*-O-4 cleavage and GC–MS demonstrated the peak abundance of vanillic acid (compound 11) increased with the H_2_O_2_ and Mn^2+^ addition. Proteomics results detected the Dyp-type peroxidase (PP_3248) in the secretome. Therefore, the *β*-O-4 linkage was degraded by Dyp-type peroxidase, forming vanillic acid as the degradation product (Fig. 5).

NMR results revealed the presence of secretome alone with lignin degraded the resinol linkage (*β*-*β*). Based on the homologous alignment of the amino acid sequence in NCBI database (blastp), FAD-binding oxidoreductase (PP_5154) exhibited the 62.8% similarity to pinoresinol *α*-hydroxylase in *Pseudomonas sp.* SG-MS2 [48]. Therefore, resinol linkage might be converted to vanillin and vanillic acid by FAD-binding oxidoreductase (Fig. 5). Besides, the addition of H_2_O_2_ stimulated the resinol linkage cleavage, suggesting the peroxidase might be involved in *β*-*β* bond degradation. Therefore, peroxidase might be involved in the resinol linkage cleavage and forming vanillic acid as the intermediate (Fig. 5).

NMR results revealed the presence of secretome alone with lignin degraded the phenylcoumaran linkage (*β*-*5*). Notably, choline dehydrogenase (betA) exhibited 40% of similarity to phcC/D in *Sphingobium sp.* SYK-6 based on blastp analysis in NCBI for *P. putida* KT2440 [44, 49, 50]. Proteomics results demonstrated the aldehyde dehydrogenase (aldA/aldB-I) presented in *P. putida* secretome (Additional file 2: Table S4). However, lignostilbene *α*,*β*-dioxygenase (lsdD) in *Sphingobium sp.* SYK-6 did not exhibit the homology protein in *P. putida* after the blast analysis. There might be other degradation mechanisms in *P. putida* KT2440 for stilbene structure which require future investigation. Therefore, *β*-5 linkage degradation pathway of *P. putida* KT2440 is only partially consistent with the route of *Sphingobium sp.* SYK-6 (Fig. 5) [44, 49, 50]. In addition, the presence of H_2_O_2_ only slightly increased the *β*-5 linkage compared to secretome alone, suggesting the peroxidase might not involve in *β*-5 cleavage.

NMR results demonstrated the secretome alone contributed to spirodienone linkage cleavage, suggesting the *β*-1 bond cleavage capacity in the secretome. NCBI blast revealed there was no homologous protein for lsdE and lsdA in *P. putida* KT2440 [41].Therefore, there might be other mechanisms for *β*-1 linkage in *P. putida*. Besides, the presence of H_2_O_2_ stimulating the *β*-1 cleavage, and Mn^2+^ addition further enhanced β-1 cleavage, suggesting the peroxidase might be involved in *β*-1 cleavage as well. Therefore, the *β*-1 linkage degradation pathway for *P. putida* KT2440 is presented in Fig. 5.

## Discussion

Overall, the extracellular lignin degradation mechanism has been revealed by *P. putida* KT2440 via secretomic analysis. The enzymatic assay demonstrated that the secretome contains active oxidase and peroxidase. The following lignin degradation rate analysis proved that the secretome could degrade 8.6% of the lignin, and the addition of H_2_O_2_ and Mn^2+^ would increase the lignin degradation rate to 14.5%. C–C bond cleavage was observed by 2D NMR in secretome alone treatment, and *β*-O-4 bond and C–C bond (*β*-*β* and *β*-1) cleavage were elevated when H_2_O_2_ was introduced to secretome due to the activated peroxidase system. Further Mn^2+^ addition enhanced *β*-O-4 and C–C bond (*β*-*β* and *β*-1) cleavage. GC–MS results reinforced the NMR results by demonstrating elevated peak areas of aromatic monomers, such as vanillin and vanillic acid were correlated with C–C and C–O bonds cleavage. Proteomics results revealed the different groups of oxidoreductases involved in lignin degradation, and the degradation pathways were proposed.

The bacterial lignin degradation process requires extensive reducing power and energy to cope with the polymeric structure and corresponding oxidative stress during aromatic compound catabolism [34]. Therefore, many studies chose to co-fermentation lignin with nutrient-rich substrates like glucose [17, 33, 35, 51]. Our results demonstrated that the secretome harbored active oxidase, suggesting the presence of multicopper oxidase in secretome generated from *P. putida* grown on glucose. Moreover, active peroxidase was also observed in secretome, suggesting that besides Dyp-type peroxidase, other potential peroxidases are also involved in lignin catabolism. Proteomics analysis revealed that the secretome contained multicopper oxidase (CopA) and Dyp-type peroxidase (PP_3248) and presented abundant oxidoreductase enzymes. Therefore, our results demonstrated that *P. putida* also secreted active ligninolytic enzymes into extracellular space when grown on nutrient-rich media (e.g., glucose).

Our results showed that secretome alone exhibited limited lignin degradation capacity, which was lower than in the *P. putida* KT2440 strain. Moreover, our results further demonstrated that the addition of H_2_O_2_ and Mn^2+^ can activate peroxidases and increase lignin degradation, suggesting that the synergistic effect between bacteria and the enzyme system could be enhanced through chemical addition on lignin degradation. H_2_O_2_ is the oxidant and electron acceptor for bacterial peroxidase during the enzymatic reaction. Purified dye-decoloring peroxidase from *Rhodococcus* and *Pseudomonas* (e.g., Rh_Dyp, and PpDyp) revealed that the addition of Mn^2+^ in the presence of H_2_O_2_ further enhanced the enzyme activity [31, 52–54]. These results point to an alternative way to enhance the bacterial lignin bioconversion efficiency by introducing H_2_O_2_ and Mn^2+^ to work together with the enzyme system and bacteria cells.

NMR results showed secretome alone presented extensive C–C bond cleavage and limited C–O bond cleavage, suggesting the function of oxidases in the secretome. Bacteria oxidases (e.g., multicopper oxidase) are laccase-like oxidase and exhibit the C–C bond cleavage. Our results demonstrated that vanillic acid (compound 11) peak area was presented in secretome + lignin treatments (Additional file 1: Fig. S4), suggesting the involvement of multicopper oxidases in lignin degradation through Cα–Cβ cleavage [40].

The addition of H_2_O_2_ with secretome elevated the *β*-O-4 bond cleavage of lignin which might be attributed to the activation of dye-decoloring peroxidase (Dyp) (Table 1). Our results identified the syringaldehyde like compounds such as phenol, 2,6-dimethoxy-4-(2-propenyl)- (compound 14) and ethanone, 1-(4-hydroxy-3,5-dimethoxyphenyl)- (compound 16), which were presented limited amount in secretome treatments but elevated when lignin was treated with H_2_O_2_. Besides, our results showed the phenolic products (compounds 3 and 9) were elevated when H_2_O_2_ was presented with secretome, suggesting the possible non-enzymatic cleavage for the phenolic type of *β*-O-4 bond [54].

NMR results also revealed secretome alone presented a series of C–C bond cleavages including *β*-*β*, *β*-5, and *β*-1 (Table 1 and Fig. 5). Our results revealed the *β*-*β* linkage was initially catalyzed via *α*-hydroxylation by vanillyl alcohol oxidase/p-cresol methyl hydroxylase (VAO/PCMH) like enzyme (PP_5154), forming vanillin and vanillic acid as the intermediate compounds during the degradation process [48, 55]. Our results showed *β*-5 linkage was cleavage by choline dehydrogenase (betA) and aldehyde dehydrogenase (aldA/aldB-I) which was partially consistent with literature [56]. In addition, no homologous proteins for lsdE and lsdA were found in *P. putida* KT2440 showed there might be a different *β*-1 linkage breakdown mechanism compared to Gram-negative bacteria *Novosphingobium aromaticivorans* DSM12444 [41].

Apart from the compounds that were confirmed by previous literature, we also identified some aromatic dimer compounds in different treatments. For example, [1,1'-biphenyl]-3,3'-dicarboxaldehyde, 6,6'-dihydroxy-5,5'-dimethoxy- (compound 30) which might be correlated with 5–5’ linkage cleavage and 3-(4-acetoxy-3-methoxyphenyl)-7-methoxy-4-oxo-4H-chromene (compound 26) might be related to tricin cleavage. However, more compounds were not correlated with specific reaction mechanisms that showed more reactions in the lignin degradation process. Our results also demonstrated inconsistency in secretome treatments with a lower aromatic compound release but larger linkage cleavage than H_2_O_2_ treatment, implying the low aromatic compounds released might be another bottleneck in the bacterial lignin degradation process. Future studies can focus on engineering oxidative degradation enzymes towards a higher  aromatic compounds release.

Proteomics-guided systems-biology approaches have been proven to discover potential pathways involved in lignin catabolism effectively [57]. Previously Dyp only reportedly appeared overexpressed in exoproteome of *P. putida* from the lignin-rich media [33]. However, our secretomic analysis revealed that Dyp, SOD, CopA, and other accessory enzymes such as catalase, dehydrogenases, reductases, dioxygenases, and oxidases all existed in the secretome from glucose which was different from the previously reported lignin inducing hypothesis. Overall, the extracellular lignin degradation pathways by *P. putida* are not well understood, primarily due to their broad prospects under varied environmental conditions [21, 27, 37, 40–46]. This distinctive study provided detailed information on lignin degradation products and oxidoreductase enzymes, resulting in new insights into the lignin depolymerization pathways in *P. putida* KT2440 for future metabolic engineering design to improve lignin bioconversion efficiency.

## Conclusions

In summary, based on enzyme assay, GC–MS, NMR, and proteomics analysis, the extracellular lignin degradation mechanisms of the secretome from *P. putida* KT2440 were elucidated as follows: (1) oxidase (e.g., multicopper oxidase, CopA) exhibited limited *β*-Ο-4 bond cleavage capacity; (2) peroxidase (e.g., dye-decoloring peroxidase, DyP) was activated by H_2_O_2_ and enhanced by Mn^2+^ addition, stimulating the *β*-Ο-4, *β*-*β*, and *β*-1 bond cleavage; (3) degradation reaction mechanisms involved in Cα–Cβ cleavage, Cα-oxidation, Cα-hydroxylation, followed by aromatic intermediates release (e.g., vanillin and vanillic acid); (4) abundant oxidoreductase enzymes in *P. putida* secretome participated in lignin degradation. To be specific, Dyp-type peroxidase, multicopper oxidase, NAD(P)H dehydrogenase, glutathione reductase, glutathione S-transferase, and choline dehydrogenase participated in *β*-Ο-4 bond cleavage. Results showed that FAD-binding oxidoreductase involved in *β-β* bond cleavage, choline dehydrogenase and aldehyde dehydrogenase involved in *β*-5 bond cleavage, and peroxidases involved in *β*-*β* and *β*-1 bond cleavage. Overall, our study provides a comprehensive understanding and list of functional enzymes of the extracellular lignin degradation pathway in *P. putida* KT2440 in order to build a roadmap for future metabolic engineering design to improve the efficiency of lignin bioconversion. Further research can be carried out to overexpress identified functional proteins for specific lignin degradation mechanism studies.

## Methods

### Microorganism and medium

*Pseudomonas putida* KT2440 was purchased from American Type Culture Collection (ATCC 47054) and stored in 25% glycerol at − 80 °C. All the chemical reagents were purchased from Sigma Aldrich and Fisher Scientific with ACS grade (99% purity). The composition of M9 mineral medium was as follows: 6 g·L^−1^ Na_2_HPO_4_, 5 g·L^−1^ glucose, 3 g·L^−1^ KH_2_PO_4_, 1 g·L^−1^ NH_4_Cl, 0.5 g·L^−1^ NaCl, 0.12 g·L^−1^ MgSO_4_ and 1 mL·L^−1^ 1000-fold trace element mix solution to make the final concentrations as follows: 3 μM (NH_4_)_6_Mo_7_O_24_·4H_2_O, 0.4 mM H_3_BO_3_, 30 μM CoCl_2_·6H_2_O, 10 μM CuSO_4_·5H_2_O, 80 μM MnCl_2_·4H_2_O, 10 μM ZnSO_4_·7H_2_O, 1 μM FeSO_4_·7H_2_O [38, 58, 59].

### Protein concentration measurement

1 mL of fermentation broth was taken from each M9 medium culture every 24 h of fermentation with two replicates. The supernatant was separated by centrifuge at 8014 g (8000 rpm) for 5 min. Parallelly, another 1 mL cell culture was taken from each M9 medium for total protein measurement. Cell cultures were frozen at -80 °C for 15 min at first. The cell suspension was taken into a water bath (42 °C) for 5 min and repeated the freeze–thaw cycle three times. The supernatant and total protein concentrations were estimated by the Pierce™ BCA protein assay kit (Thermo Scientific, San Jose, CA).

### Ligninolytic activity assays for intracellular and secretome

A 1 mL cell culture was taken from each M9 medium culture at different fermentation times and stored in a 1.5-mL centrifuge tube for total enzyme activity assay measurement (intracellular + secretome) with two replicates. Cell cultures were frozen under -80 °C for 15 min at first. The cell suspension was taken into a water bath (42 °C) for 5 min and repeated the freeze–thaw cycle three times. Another 1 mL cell culture was also taken from each M9 medium culture and centrifuged at 8000 rpm for 5 min. Then, the supernatant was transferred to a new 1.5-mL centrifuge tube for secretome enzyme activity assay measurement. Laccase and peroxidase activity were examined daily in the culture supernatants by the oxidation of 5 mM DMP (synonym syringol) to dimeric cerulignone (ε469 = 55 000 M^−1^ cm^−1^) in 0.1 mM sodium malonate buffer at pH 7. For peroxidase activity assays, 0.1 mM H_2_O_2_ was also added to initiate the reaction. In addition, the latter assays were performed in the absence and the presence of Mn^2+^ (0.1 mM MnSO_4_). Absorbance from peroxidase activity was corrected with that caused by laccase activity. Measurements were carried out at room temperature. One unit (1 U) of activity is defined as the amount of enzyme releasing 1 μmol of product per minute under the defined reaction conditions [17].

### Secretome harvest

*P. putida* strains were fed in 100 mL M9 medium in a 250-mL flask with 5 g·L^−1^ glucose and 1 g·L^−1^ NH_4_Cl for 5 days. The fermentation broth was transferred to a couple of 50 mL centrifuge tubes. The supernatant and cells were separated by centrifugation at 8014 g (8000 rpm) for 5 min. The supernatant was transferred to a new pre-cooled flask (1 L) in ice batch. The supernatant was centrifuged again at 8014 g (8000 rpm) for 5 min and then passed through 0.22 μm sterilized filter (Millipore® Stericup® filtration system) to a new flask (1 L) for further degradation experiment. Ligninolytic enzyme activities and protein concentration were measured prior to the lignin degradation reaction.

### Lignin degradation reaction with secretome

Corn stover alkali lignin was purified following a published methodology (more details about the alkali lignin purifying process, composition and structural analysis can be found elsewhere) [60]. A fresh stock alkali lignin solution (20 g·L^−1^) was prepared at pH 12.5 and then adjusted to pH 7. 250 mL Erlenmeyer flasks with 50 mL of lignin degradation reaction solution were cultivated in a shaking incubator at 180 rpm and set to 37 °C. The basic composition of lignin degradation reaction solution (50 mL) was as follows: secretome solution 45 mL, lignin stock solution 5 mL (100 mg, final concentration 2 g·L^−1^), and sodium malonate 0.1 mM. The reaction solution was also supplemented with H_2_O_2_ (0.1 mM) and Mn^2+^ (0.1 mM) to evaluate their effect on lignin degradation together with secretome. The secretome or lignin control contained 45 mL secretome solution with 5 mL Milli-Q water or 5 mL lignin stock solution with 45 mL Milli-Q water. Every treatment was a set of three replicates with a total of 18 flasks.

The reaction was stopped after 120 h, and the reaction solution was transferred to a 50-mL centrifuge tube. The lignin amount determination followed NREL Laboratory Analytical Procedures (LAPs) [61, 62]. The reaction solution was first adjusted pH to 2 by adding the hydrochloric acid (1 mol·L^−1^). The acid-insoluble lignin was harvested by centrifugation at 8014 g (8000 rpm) for 5 min. The supernatant was carefully transferred to a new 50 mL centrifuge tube for degradation products analysis. The acid-insoluble lignin was freeze-dried and weighed. The acid-insoluble lignin from secretome treatments was corrected with the acid-precipitated and weighed secretome control to remove the protein amount. The lignin degradation rate was indicated as the difference between the remaining lignin after different treatments and control, followed by dividing the control [17].

### Analysis of lignin degradation products

The lignin degradation products in the reaction solution were determined by gas chromatography–mass spectroscopy (GC–MS). Every treatment was a set of three replicates. 15 mL of ethyl acetate was added to 15 mL solutions from the different samples in a 50-mL centrifuge tube and was vortexed for 5 min at room temperature. The top ethyl acetate layer was then transferred to a glass tube. The remained reaction solution was extracted again with 15 mL ethyl acetate and the two extracts were merged. The glass tubes were left in the fume hood for 7 days to let the ethyl acetate evaporate naturally to around 2 mL. The ethyl acetate was concentrated to around 1 mL by passing nitrogen for 20 min and then filtered with 0.45 μm PTFE membrane into the GC vials [63]. The organic solvent-extracted samples (1 μL) were injected into a stream of He (carrier gas) flowing at 1.2 mL·min^−1^ into a DB5 (30 m × 0.250 μm × 0.25 μm) capillary column fitted in an Agilent Technologies 7890A GC system set in the splitless mode. The GC oven was programmed to reach 45 °C and maintained this temperature for 2 min; then ramp up at the rate of 15 °C min^−1^ until the temperature reached 200 °C, and held at this temperature for 1 min, after which the temperature was increased at a rate of 5 °C min^−1^ until the temperature reached 280 °C. At this temperature, it was held for 7 min. Eluting compounds were detected with an MS (Agilent Technologies 5975C) inert XL EI/CI MSD with a triple-axis detector and compared using NIST libraries [64].

### 2D HSQC NMR analysis

2D-^1^H–^13^C heteronuclear single-quantum coherence (HSQC) nuclear magnetic resonance (NMR) spectra were obtained using a Bruker Avance III HD 500 MHz spectrometer operating at a frequency of 125.12 MHz for the ^13^C nucleus. 30–50 mg of the dry lignin samples were dissolved in 0.6 mL deuterated dimethylsulfoxide (DMSO)-*d*_6_ and the spectra were collected at 298 K. A standard Bruker adiabatic HSQC pulse sequence (hsqcetgpsisp2.2) was used with the following spetra acquisition condition: 1.0 s pulse delay, 64 scans, 1024 data points for ^1^H, 256 increments for ^13^C, and a ^1^*J*_C–H_ of 145 Hz. The ^1^H and ^13^C spectral widths are 13.0 and 220.0 ppm, respectively. The central DMSO solvent peak (δ^13^C/δ^1^H = 39.5/2.49 ppm) was used for chemical shifts calibration. HSQC spectra were processed and analyzed with Mestrenova (version 12.0.2) with a matched cosine-bell apodization and 2 × zero filling in both dimensions. The content of the inter-linkages are expressed as a percentage relative to the total lignin subunits (G + H + S) [65].

### Protein extraction and tryptic digestion

*P. putida* strains were fed in 100 mL M9 medium in a 250 mL flask with 5 g·L^−1^ glucose and 1 g·L^−1^ NH_4_Cl. The experiment was conducted with four replicates. The cells and supernatant were harvested at 120 h of fermentation. The fermentation broth was centrifuged at 8014 g (8000 rpm) for 5 min using Eppendorf 5804 to separate the supernatant and cell pellet. The cell pellet was washed twice with 5 mL of 0.9% sodium chloride solution. The supernatant was filtered with 0.22-μm PTFE membrane into new 50-mL centrifuge tubes. Cell pellet and supernatant were stored at − 80 °C fridge for further protein extraction.

Cell pellets were stored in regular 1.5-mL centrifuge tubes. Cell pellets were resuspended in a 250 μL lysis buffer solution (8 M urea, 75 mM NaCl in 100 mM NH_4_HCO_3_, pH 7.8), transferred to a 1.5-mL safe-lock centrifuge tube. A scoop of zirconia/silica beads (~ 100 μL) was added to each tube, and the bead beating experiment was performed by 8 rounds of 30 s using a Bullet Blender (Homogenizers, Atkinson, NH) [66]. After bead beating, a needle was used to poke a hole at the bottom of the 1.5-mL tube and put on a 15-mL falcon tube to collect the supernatant by centrifugation at 2000 rpm, 4 °C for 5 min. The beads were then washed with 100 µL lysis buffer and centrifuged at 2000 rpm at 4 °C for 5 min. The lysate was then transferred to a new 2-mL centrifuge tube and pellet the cellular debris at 14,000 rpm for 10 min at 4 °C. The supernatant was transferred to a new 2-mL centrifuge tube for further protein digestion procedures.

Parallelly, 20 mL of supernatant was collected, and the proteins were first denatured by adding 15 g urea (final concentration 8 M) and incubating for 1 h at 37 °C. The supernatant was then concentrated using a 30-kDa filter (EMD Millipore, Billerica, MA) by centrifugation at 4000 rpm, 4 °C for 30 min. The concentrated supernatant was transferred to a clean 2-mL tube for further protein digestion procedures.

The protein purification and digestion of intracellular protein and supernatant samples were conducted with the FASP Protein Digestion Kit (Expedeon, San Diego, CA) with trypsin (Promega, Madison, WI) following the manufacturer’s instruction. The protein concentration was estimated by the Pierce™ BCA protein assay (Thermo Scientific, San Jose, CA) and normalized to 0.1 μg μL^−1^ before LC–MS/MS analysis. Four biological replicates were applied during the entire process [34].

### Proteomic data acquisition and analysis

LC–MS/MS analysis was performed using an Orbitrap Fusion Lumos mass spectrometer (Thermo Scientific, San Jose, CA). Tryptic peptide digests were separated using a nanoACQUITY UPLC systems (Waters, Milford, MA) by reversed-phase HPLC with 110 min gradient time at a column flow rate of 200 nL/min. The detailed equipment parameters setup was described in a recent publication by Wang et al. [67] In terms of proteomic data analysis, raw MS/MS data files were processed with MaxQuant (version 1.6.7.0). After loading all the raw data and giving appropriate names, label-free quantification (LFQ) algorithm was used with a minimum LFQ ratio count of 2 for relative quantification in the Group-specific parameters section. Trypsin was selected for digestion mode with a maximum of two missed cleavages. The peptide tandem mass spec raw data were searched against the Uniport FASTA files of strain *P. putida* KT2440 (released at 07, April 2017, Taxonomy ID: 160488). In the global parameters section, the second peptides and match between runs features were enabled with a 0.7-min match time window and 20-min alignment time window. The spectral level false discovery rate (FDR, q value) was < = 1% based on a decoy search [68]. Other parameters just followed the default settings. The protein intensities obtained from Maxquant software were log2 transformed. The Venn chart was generated by Venn-Diagram-Plotter (version 1.6.7458, https://github.com/PNNL

-Comp-Mass-Spec/Venn-Diagram-Plotter/releases/tag/v1.6.7458). Pie chart was generated by Origin software (version 9.8.5.204).

## Supplementary Information

**Additional file 1: Figure S1.** The overall workflow for identifying bacterial lignin degradation pathways. **Figure S2.** GC–MS analysis of lignin breakdown products. **Figure S3.** Lignin breakdown products identified by GC–MS. **Figure S4.** The variation of peaks area of all lignin breakdown products. **Figure S5.** Main detected lignin linkages. **Table S1.** Occurrence of putative degradation products of lignin in different treatment conditions. **Table S2.** Grouped lignin breakdown products among all treatments. **Table S3.** Main lignin 2D ^1^H–^13^C Cross-peak assignments in the HSQC Spectra.

**Additional file 2: Table S4.** Proteomic analysis of the intracellular and secretome in *P. putida* KT2440.

**Additional file 3.** A list of abbreviations is included.

## Data Availability

All data generated or analyzed during this study are included in this published article and its supplementary information files.

## References

[1] Wang H, Pu Y, Ragauskas A, Yang B (2019). From lignin to valuable products-strategies, challenges, and prospects. Bioresour Technol.

[2] Ragauskas AJ, Beckham GT, Biddy MJ, Chandra R, Chen F, Davis MF, Davison BH, Dixon RA, Gilna P, Keller M (2014). Lignin valorization: improving lignin processing in the biorefinery. Science.

[3] Laskar DD, Yang B, Wang HM, Lee J (2013). Pathways for biomass-derived lignin to hydrocarbon fuels. Biofuel Bioprod Biorefin.

[4] Liu Z-H, Hao N, Wang Y-Y, Dou C, Lin F, Shen R, Bura R, Hodge DB, Dale BE, Ragauskas AJ (2021). Transforming biorefinery designs with ‘Plug-in processes of lignin’ to enable economic waste valorization. Nat Commun.

[5] Liu Z-H, Le RK, Kosa M, Yang B, Yuan J, Ragauskas AJ (2019). Identifying and creating pathways to improve biological lignin valorization. Renew Sust Energ Rev.

[6] Laaksometsä C, Axelsson E, Berntsson T, Lundström A (2009). Energy savings combined with lignin extraction for production increase: case study at a eucalyptus mill in Portugal. Clean Technol Environ Policy.

[7] Yang B, Tao L, Wyman CE (2018). Strengths, challenges, and opportunities for hydrothermal pretreatment in lignocellulosic biorefineries. Biofuels Bioprod.

[8] Sethupathy S, Murillo Morales G, Gao L, Wang H, Yang B, Jiang J, Sun J, Zhu D (2022). Lignin valorization: status, challenges and opportunities. Bioresour Technol.

[9] Rizzi F, van Eck NJ, Frey M (2014). The production of scientific knowledge on renewable energies: Worldwide trends, dynamics and challenges and implications for management. Renew Energy.

[10] Li X, Weng JK, Chapple C (2008). Improvement of biomass through lignin modification. Plant J.

[11] Dai J, Patti AF, Saito K (2016). Recent developments in chemical degradation of lignin: catalytic oxidation and ionic liquids. Tetrahedron Lett.

[12] Higuchi T (2004). Microbial degradation of lignin: role of lignin peroxidase, manganese peroxidase, and laccase. Proc Jpn Acad, Ser B.

[13] Brown ME, Chang MC (2014). Exploring bacterial lignin degradation. Curr Opin Chem Biol.

[14] Wang H, Yang B, Zhang Q, Zhu W (2019). Catalytic routes for the conversion of lignocellulosic biomass to aviation fuel range hydrocarbons. Renew Sust Energ Rev.

[15] Kirk TK, Farrell RL (1987). Enzymatic" combustion": the microbial degradation of lignin. Annu Rev Microbiol.

[16] Zhao Z-M, Zhang S, Meng X, Pu Y, Liu Z-H, Ledford WK, Kilbey SM, Li B-Z, Ragauskas AJ (2021). Elucidating the mechanisms of enhanced lignin bioconversion by an alkali sterilization strategy. Green Chem.

[17] Salvachua D, Karp EM, Nimlos CT, Vardon DR, Beckham GT (2015). Towards lignin consolidated bioprocessing: simultaneous lignin depolymerization and product generation by bacteria. Green Chem.

[18] Beckham GT, Johnson CW, Karp EM, Salvachúa D, Vardon DR (2016). Opportunities and challenges in biological lignin valorization. Curr Opin Biotechnol.

[19] Zhang L, Xu Z, Cort JR, Vuorinen T, Yang B (2020). Flowthrough Pretreatment of Softwood under water-only and alkali conditions. Energy Fuels.

[20] Zhang L, Pu Y, Cort JR, Ragauskas AJ, Yang B (2016). Revealing the molecular structural transformation of hardwood and softwood in dilute acid flowthrough pretreatment. ACS Sustain Chem Eng.

[21] Zhu D, Qaria MA, Zhu B, Sun J, Yang B (2022). Extremophiles and extremozymes in lignin bioprocessing. Renew Sust Energ Rev.

[22] Zhu D, Xu L, Sethupathy S, Si H, Ahmad F, Zhang R, Zhang W, Yang B, Sun J (2021). Decoding lignin valorization pathways in the extremophilic *Bacillus ligniniphilus* L1 for vanillin biosynthesis. Green Chem.

[23] Shen R, Tao L, Yang B (2018). Techno-economic analysis of jet-fuel production from biorefinery waste lignin. Biofuel Bioprod Biorefin.

[24] Cragg SM, Beckham GT, Bruce NC, Bugg TDH, Distel DL, Dupree P, Etxabe AG, Goodell BS, Jellison J, McGeehan JE (2015). Lignocellulose degradation mechanisms across the tree of life. Curr Opin Chem Biol.

[25] Erickson E, Bleem A, Kuatsjah E, Werner AZ, DuBois JL, McGeehan JE, Eltis LD, Beckham GT (2022). Critical enzyme reactions in aromatic catabolism for microbial lignin conversion. Nat Catal.

[26] Hatakka A (1994). Lignin-modifying enzymes from selected white-rot fungi: production and role from in lignin degradation. FEMS Microbiol Rev.

[27] Bugg TD, Ahmad M, Hardiman EM, Rahmanpour R (2011). Pathways for degradation of lignin in bacteria and fungi. Nat Prod Rep.

[28] ten Have R, Teunissen PJ (2001). Oxidative mechanisms involved in lignin degradation by white-rot fungi. Chem Rev.

[29] de Gonzalo G, Colpa DI, Habib MH, Fraaije MW (2016). Bacterial enzymes involved in lignin degradation. J Biotechnol.

[30] Chandra R, Raj A, Purohit HJ, Kapley A (2007). Characterisation and optimisation of three potential aerobic bacterial strains for kraft lignin degradation from pulp paper waste. Chemosphere.

[31] Ahmad M, Roberts JN, Hardiman EM, Singh R, Eltis LD, Bugg TD (2011). Identification of DypB from *Rhodococcus jostii* RHA1 as a lignin peroxidase. Biochem.

[32] Sharma P, Goel R, Capalash N (2007). Bacterial laccases. World J Microbiol Biotechnol.

[33] Salvachúa D, Werner AZ, Pardo I, Michalska M, Black BA, Donohoe BS, Haugen SJ, Katahira R, Notonier S, Ramirez KJ (2020). Outer membrane vesicles catabolize lignin-derived aromatic compounds in *Pseudomonas putida* KT2440. Proc Natl Acad Sci USA.

[34] Li X, He Y, Zhang L, Xu Z, Ben H, Gaffrey MJ, Yang Y, Yang S, Yuan JS, Qian W-J (2019). Discovery of potential pathways for biological conversion of poplar wood into lipids by co-fermentation of *Rhodococci* strains. Biotechnol Biofuels.

[35] Linger JG, Vardon DR, Guarnieri MT, Karp EM, Hunsinger GB, Franden MA, Johnson CW, Chupka G, Strathmann TJ, Pienkos PT (2014). Lignin valorization through integrated biological funneling and chemical catalysis. Proc Natl Acad Sci USA.

[36] Salvachua D, Katahira R, Cleveland NS, Khanna P, Resch MG, Black BA, Purvine SO, Zink EM, Prieto A, Martinez MJ (2016). Lignin depolymerization by fungal secretomes and a microbial sink. Green Chem.

[37] Granja-Travez RS, Bugg TDH (2018). Characterization of multicopper oxidase CopA from *Pseudomonas putida* KT2440 and *Pseudomonas fluorescen*s Pf-5: involvement in bacterial lignin oxidation. Arch Biochem Biophys.

[38] Lin L, Cheng Y, Pu Y, Sun S, Li X, Jin M, Pierson EA, Gross DC, Dale BE, Dai SY (2016). Systems biology-guided biodesign of consolidated lignin conversion. Green Chem.

[39] del Cerro C, Erickson E, Dong T, Wong AR, Eder EK, Purvine SO, Mitchell HD, Weitz KK, Markillie LM, Burnet MC (2021). Intracellular pathways for lignin catabolism in white-rot fungi. Proc Natl Acad Sci USA.

[40] Rashid GMM, Bugg TDH (2021). Enhanced biocatalytic degradation of lignin using combinations of lignin-degrading enzymes and accessory enzymes. Catal Sci Technol.

[41] Presley GN, Werner AZ, Katahira R, Garcia DC, Haugen SJ, Ramirez KJ, Giannone RJ, Beckham GT, Michener JK (2021). Pathway discovery and engineering for cleavage of a β-1 lignin-derived biaryl compound. Metab Eng.

[42] Lin L, Wang X, Cao L, Xu M (2019). Lignin catabolic pathways reveal unique characteristics of dye-decolorizing peroxidases in *Pseudomonas putida*. Environ Microbiol.

[43] Chen Z, Wan C (2017). Biological valorization strategies for converting lignin into fuels and chemicals. Renew Sust Energ Rev.

[44] Naofumi K, Kenji T, Kosuke M, Takuma A, Masaya F, Yudai H, Eiji M (2017). Bacterial catabolism of lignin-derived aromatics: new findings in a recent decade: update on bacterial lignin catabolism. Environ Microbiol Rep.

[45] Kamimura N, Sakamoto S, Mitsuda N, Masai E, Kajita S (2019). Advances in microbial lignin degradation and its applications. Curr Opin Biotechnol.

[46] Sainsbury PD, Hardiman EM, Ahmad M, Otani H, Seghezzi N, Eltis LD, Bugg TDH (2013). Breaking down lignin to high-value chemicals: the conversion of lignocellulose to vanillin in a gene deletion mutant of *Rhodococcus jostii* RHA1. ACS Chem Biol.

[47] Prates ET, Crowley MF, Skaf MS, Beckham GT (2019). Catalytic mechanism of aryl-ether bond cleavage in lignin by LigF and LigG. J Phys Chem B.

[48] Shettigar M, Balotra S, Kasprzak A, Pearce SL, Lacey MJ, Taylor MC, Liu J-W, Cahill D, Oakeshott JG, Pandey G (2020). Oxidative Catabolism of (+)-Pinoresinol is initiated by an unusual flavocytochrome encoded by translationally coupled genes within a cluster of (+)-Pinoresinol-Coinduced Genes in *Pseudomonas* sp Strain SG-MS2. Appl Environ Microbiol.

[49] Kamimura N, Hirose Y, Masuba R, Kato R, Takahashi K, Higuchi Y, Hishiyama S, Masai E (2021). LsdD has a critical role in the dehydrodiconiferyl alcohol catabolism among eight lignostilbene α,β-dioxygenase isozymes in Sphingobium sp strain SYK-6. Int Biodeterior Biodegradation.

[50] Kuatsjah E, Chan ACK, Katahira R, Haugen SJ, Beckham GT, Murphy MEP, Eltis LD (2021). Structural and functional analysis of lignostilbene dioxygenases from Sphingobium sp SYK-6. J Biol Chem.

[51] Liu Z-H, Shinde S, Xie S, Hao N, Lin F, Li M, Yoo CG, Ragauskas AJ, Yuan JS (2019). Cooperative valorization of lignin and residual sugar to polyhydroxyalkanoate (PHA) for enhanced yield and carbon utilization in biorefineries. Sustain Energy Fuels.

[52] Vignali E, Tonin F, Pollegioni L, Rosini E (2018). Characterization and use of a bacterial lignin peroxidase with an improved manganese-oxidative activity. Appl Microbiol Biotechnol.

[53] Santos A, Mendes S, Brissos V, Martins LO (2014). New dye-decolorizing peroxidases from Bacillus subtilis and *Pseudomonas putida* MET94: towards biotechnological applications. Appl Microbiol Biotechnol.

[54] Singh R, Grigg JC, Qin W, Kadla JF, Murphy MEP, Eltis LD (2013). Improved manganese-oxidizing activity of DypB, a Peroxidase from a Lignolytic Bacterium. ACS Chem Biol.

[55] Shettigar M, Balotra S, Cahill D, Warden AC, Lacey MJ, Kohler H-PE, Rentsch D, Oakeshott JG, Pandey G (2018). Isolation of the (+)-pinoresinol-mineralizing *Pseudomonas sp.* strain SG-MS2 and elucidation of its catabolic pathway. Appl Environ Microbiol.

[56] Zhang K, Si M, Liu D, Zhuo S, Liu M, Liu H, Yan X, Shi Y (2018). A bionic system with Fenton reaction and bacteria as a model for bioprocessing lignocellulosic biomass. Biotechnol Biofuels.

[57] Li X, Xu Z, Gluth A, Qian W-J, Yang B, Chang GY, Arthur R (2021). Proteomic approaches for advancing the understanding and application of oleaginous bacteria for bioconversion of lignin to lipids. Lignin Utilization Strategies: From Processing to Applications.

[58] Sambrook J: Molecular cloning : a laboratory manual: Third edition. Cold Spring Harbor, NY Cold Spring Harbor Laboratory Press [2001] ©2001; 2001.

[59] Xu Z, Li X, Hao N, Pan C, de latorre L, Ahamed A, Miller JH, Ragauskas AJ, Yuan J, Yang B (2019). Kinetic understanding of nitrogen supply condition on biosynthesis of polyhydroxyalkanoate from benzoate by *Pseudomonas putida* KT2440. Bioresour Technol.

[60] Wang H, Ruan H, Pei H, Wang H, Chen X, Tucker MP, Cort JR, Yang B (2015). Biomass-derived lignin to jet fuel range hydrocarbons via aqueous phase hydrodeoxygenation. Green Chem.

[61] Sluiter A, Hames B, Ruiz R, Scarlata C, Sluiter J, Templeton D, Crocker D (2008). Determination of structural carbohydrates and lignin in biomass. Lab Anal Proced.

[62] Sluiter A, Hames B, Ruiz R, Scarlata C, Sluiter J, Templeton D (2006). Determination of sugars, byproducts, and degradation products in liquid fraction process samples. Gold Natl Renew Energy Lab.

[63] He Y, Li X, Ben H, Xue X, Yang B (2017). Lipid production from dilute alkali corn stover lignin by *Rhodococcus* strains. ACS Sustain Chem Eng.

[64] Wang H, Ruan H, Feng M, Qin Y, Job H, Luo L, Wang C, Engelhard MH, Kuhn E, Chen X (2017). One-pot process for hydrodeoxygenation of lignin to alkanes using Ru-based bimetallic and bifunctional catalysts supported on Zeolite Y. Chemsuschem.

[65] Liu Z-H, Hao N, Shinde S, Pu Y, Kang X, Ragauskas AJ, Yuan JS (2019). Defining lignin nanoparticle properties through tailored lignin reactivity by sequential organosolv fragmentation approach (SOFA). Green Chem.

[66] Aylward FO, Khadempour L, Tremmel DM, McDonald BR, Nicora CD, Wu S, Moore RJ, Orton DJ, Monroe ME, Piehowski PD (2015). Enrichment and Broad representation of plant biomass-degrading enzymes in the specialized hyphal swellings of *Leucoagaricus gongylophorus*, the fungal symbiont of leaf-cutter ants. PLoS ONE.

[67] Wang L-B, Karpova A, Gritsenko MA, Kyle JE, Cao S, Li Y, Rykunov D, Colaprico A, Rothstein JH, Hong R (2021). Proteogenomic and metabolomic characterization of human glioblastoma. Cancer Cell.

[68] Xu Z, Pan C, Li X, Hao N, Zhang T, Gaffrey MJ, Pu Y, Cort JR, Ragauskas AJ, Qian W-J (2021). Enhancement of polyhydroxyalkanoate production by co-feeding lignin derivatives with glycerol in *Pseudomonas putida* KT2440. Biotechnol Biofuels.

[69] Kumar A, Chandra R (2020). Ligninolytic enzymes and its mechanisms for degradation of lignocellulosic waste in environment. Heliyon.

