# Supplementary material for: Understanding of bacterial lignin extracellular degradation mechanisms by Pseudomonas putida KT2440 via secretomic analysis
Source: Biotechnol Biofuels Bioprod. 2022 Oct 31;15:117. doi: 10.1186/s13068-022-02214-x (PMC9620641; doi:10.1186/s13068-022-02214-x)
Supplement: Supplementary file 1 — Additional file 1: Figure S1. The overall workflow for identifying bacterial lignin degradation pathways. Figure S2. GC–MS analysis of lignin breakdown products. Figure S3. Lignin breakdown products identified by GC–MS. Figure S4. The variation of peaks area of all lignin breakdown products. Figure S5. Main detected lignin linkages. Table S1. Occurrence of putative degradation products of lignin in different treatment conditions. Table S2. Grouped lignin breakdown products among all treatments. Table S3. Main lignin 2D 1H–13C Cross-peak assignments in the HSQC Spectra. [file 13068_2022_2214_MOESM1_ESM.docx]

Understanding of Bacterial Lignin Extracellular Degradation Mechanisms by *Pseudomonas putida* KT2440 via Secretomic Analysis

Zhangyang Xu^a^, Bo Peng^a^, Reta Birhanu Kitata^b^, Carrie D. Nicora^b^, Karl K. Weitz^b^, Yunqiao Pu^c^, Tujin Shi^b^, John R. Cort^b^, Arthur J. Ragauskas^c,d,e^, and Bin Yang^a,b*^

1. Bioproducts, Sciences & Engineering Laboratory, Department of Biological Systems

Engineering, Washington State University, Richland, Washington, 99354, United States.

1. Biological Sciences Division, Pacific Northwest National Laboratory, Richland, Washington, 99352, United States.
2. Joint Institute for Biological Sciences, Biosciences Division, Oak Ridge National Laboratory, Oak Ridge, Tennessee, 37831, United States.
3. Department of Chemical and Biomolecular Engineering, University of Tennessee,

Knoxville, Tennessee, 37996, United States.

1. Department of Forestry, Wildlife, and Fisheries, Center for Renewable Carbon, University of Tennessee Institute of Agriculture, Knoxville, Tennessee, 37996, United States.

**Additional Figures and Tables**


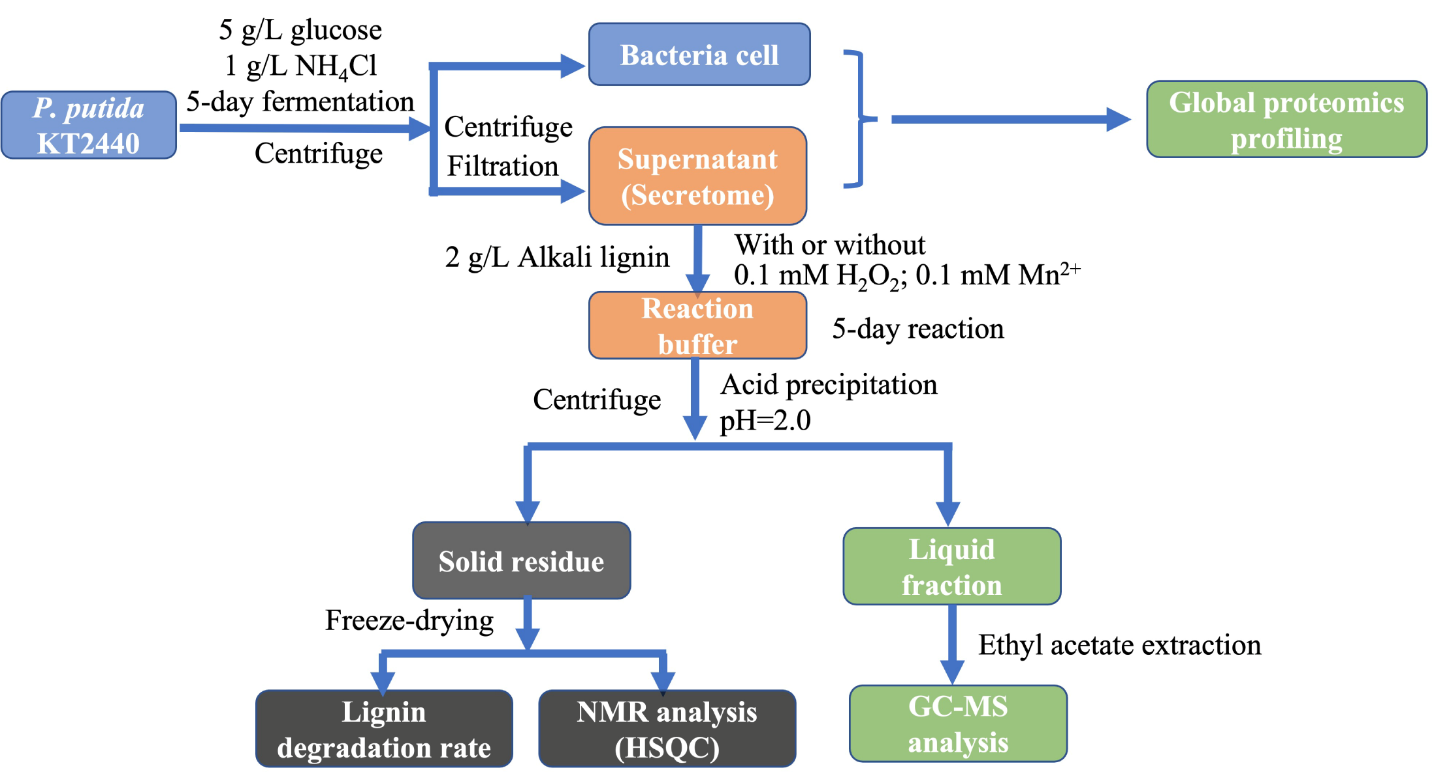


**Fig.S1.** The overall workflow for identifying bacterial lignin degradation pathways.

**
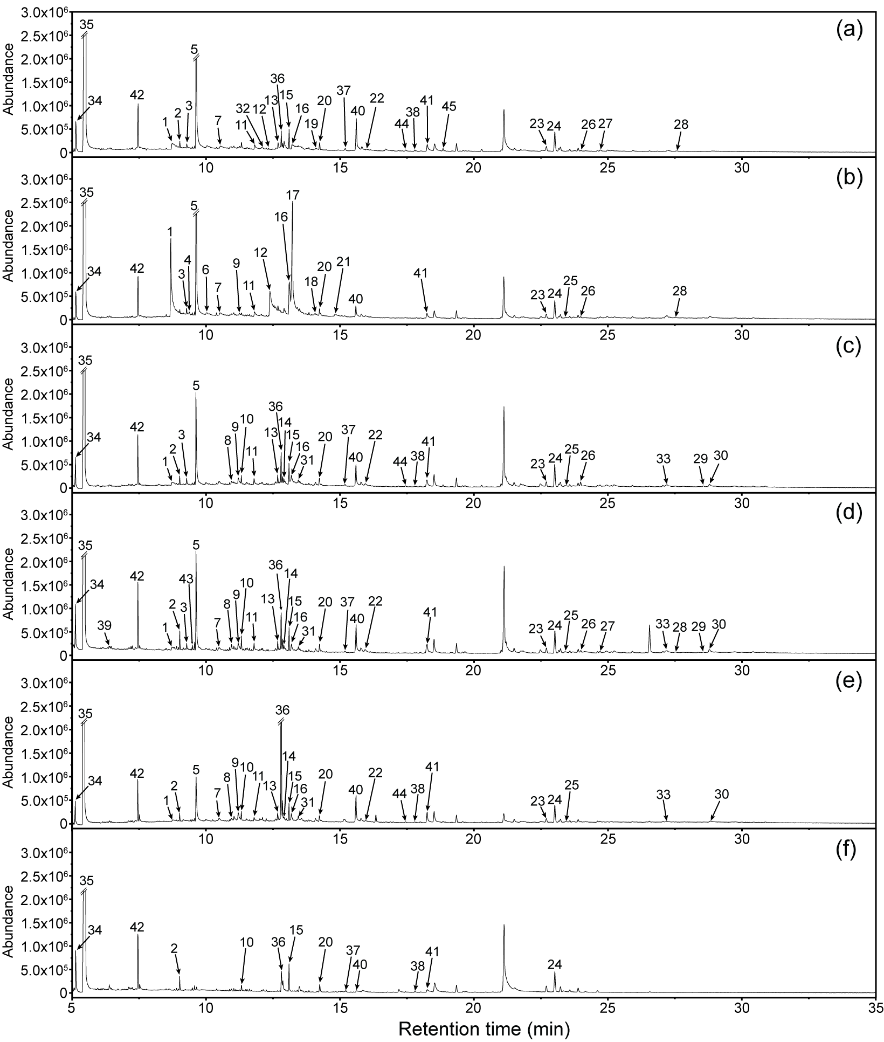
**

**Fig. S2.** GC-MS analysis of lignin breakdown products. (a) lignin control, (b) lignin + 0.1 mM H_2_O_2_, (c) lignin + secretome, (d) lignin + secretome + 0.1 mM H_2_O_2_, (e) lignin + secretome + 0.1 mM H_2_O_2_ + 0.1 mM Mn^2+^, (f) secretome control. Detailed peak assignments are listed in Table S1.


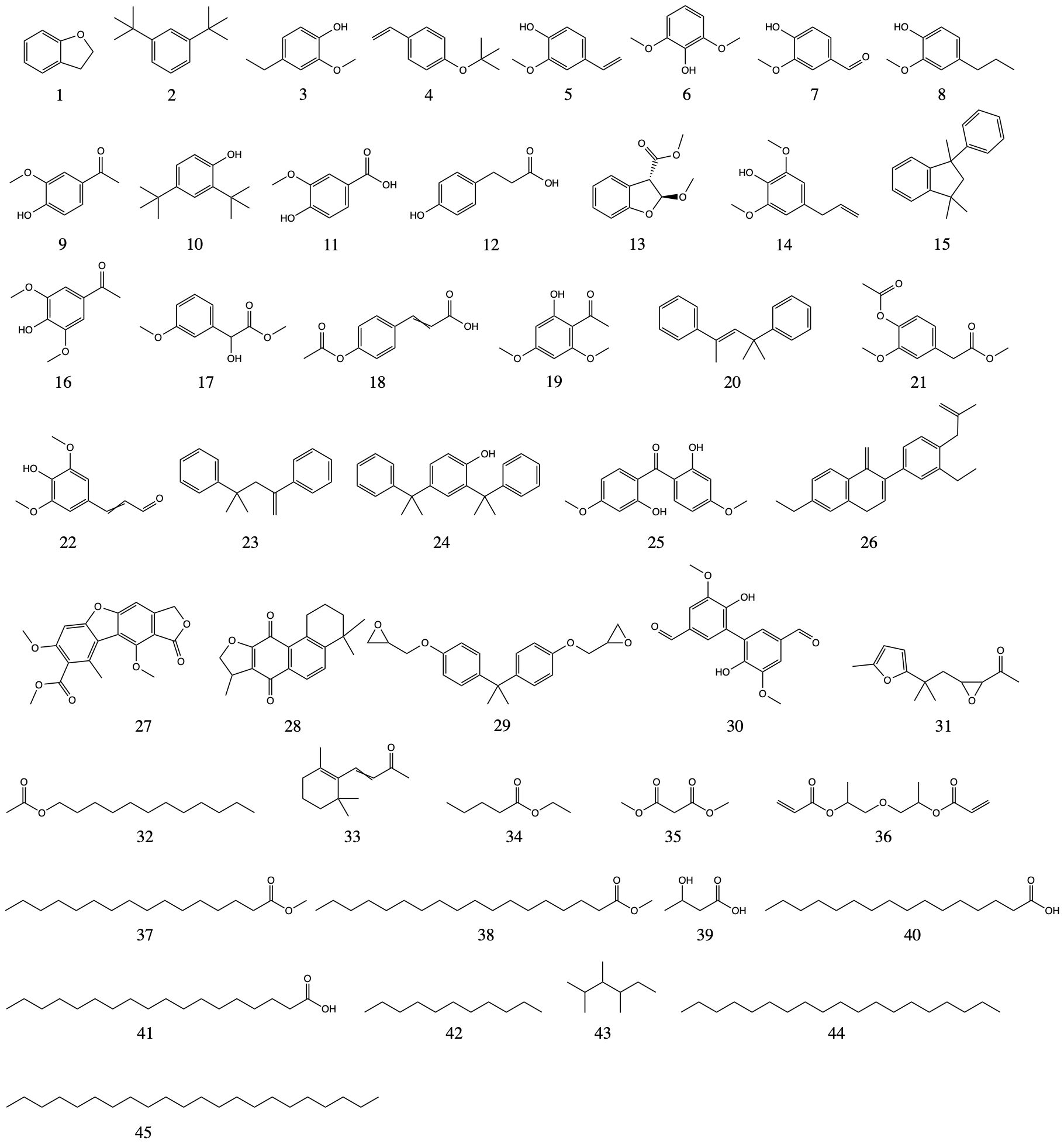


**Fig. S3.** Lignin breakdown products identified by GC-MS. The detailed information for each compound is listed in Table S1.





**Fig. S4.** The variation of peaks area of all lignin breakdown products. Lignin breakdown products were identified by GC-MS after 5 days of different treatment conditions. Lignin concentration was set as 2 g/L. H_2_O_2_ and Mn^2+^ concentration were both set as 0.1 mM. Peak numbers represent the aromatic compounds, and the detailed information are listed in Table 1. (a) peak area for peak number 1, 5, 12, 16, 17, 21, 35, 36, 42, (b) peak area for peak number 2-4, 6-11, (c) peak area for peak number 13-15, 18-20, 22-24, (d) peak area for peak number 25-33, (e) peak area for peak number 34, 37-41, 43-45. All peak areas were calculated from three replicates (n=3).

**Fig. S5.** Main detected lignin linkages.

Table S1. Occurence of putative degradation products of lignin in different treatment conditions ^a^.

| **No.** | **RT** | **Compounds** | **Treatment** | | | | | |
| --- | --- | --- | --- | --- | --- | --- | --- | --- |
|  | **min** |  | **L** | **L+H** | **S+L** | **S+L+H** | **S+L+H+M** | **S** |
| **Aromatic compounds** | | |  |  |  |  |  |  |
| 1 | 8.738 | Benzofuran, 2,3-dihydro- | + | + | + | + | + | - |
| 2 | 9.019 | Benzene, 1,3-bis(1,1-dimethylethyl)- | + | - | + | + | + | + |
| 3 | 9.289 | Phenol, 4-ethyl-2-methoxy- | + | + | + | + | - | - |
| 4 | 9.375 | 4-tert-Butoxystyrene | - | + | - | - | - | - |
| 5 | 9.634 | 2-Methoxy-4-vinylphenol | + | + | + | + | + | - |
| 6 | 10.012 | Phenol, 2,6-dimethoxy- | - | + | - | - | - | - |
| 7 | 10.497 | Vanillin | + | + | + | + | + | - |
| 8 | 10.95 | Phenol, 2-methoxy-4-propyl- | - | - | + | + | + | - |
| 9 | 11.198 | Acetovanillone | - | + | + | + | + | - |
| 10 | 11.317 | Phenol, 2,4-bis(1,1-dimethylethyl)- | - | - | + | + | + | + |
| 11 | 11.802 | Vanillic acid | + | + | + | + | + | - |
| 12 | 12.385 | Benzenepropanoic acid, 4-hydroxy- | + | + | - | - | - | - |
| 13 | 12.676 | 3-Benzofurancarboxylic acid, 2,3-dihydro-2-methoxy-, methyl ester, trans- | + | - | + | + | + | - |
| 14 | 12.914 | Phenol, 2,6-dimethoxy-4-(2-propenyl)- | - | + | + | + | + | - |
| 15 | 13.097 | 1H-Indene, 2,3-dihydro-1,1,3-trimethyl-3-phenyl- | + | - | + | + | + | + |
| 16 | 13.184 | Ethanone, 1-(4-hydroxy-3,5-dimethoxyphenyl)- | + | + | + | + | + | - |
| 17 | 13.227 | Benzeneacetic acid, .alpha.-hydroxy-3-methoxy-, methyl ester | - | + | - | - | - | - |
| 18 | 14.068 | 4-Acetoxycinnamic acid | - | + | - | - | - | - |
| 19 | 14.1 | Ethanone, 1-(2-hydroxy-4,6-dimethoxyphenyl)- | + | - | - | - | - | - |
| 20 | 14.241 | 2,4-Diphenyl-4-methyl-2(E)-pentene | + | + | + | + | + | + |
| 21 | 14.823 | Benzeneacetic acid, 4-(acetyloxy)-3-methoxy-, methyl ester | + | + | - | - | - | - |
| 22 | 15.956 | 3,5-Dimethoxy-4-hydroxycinnamaldehyde | + | - | + | + | + | - |
| 23 | 22.699 | 2,4-Diphenyl-4-methyl-1-pentene | + | + | + | + | + | - |
| 24 | 23.023 | Phenol, 2,4-bis(1-methyl-1-phenylethyl)- | + | + | + | + | + | + |
| 25 | 23.443 | Methanone, bis(2-hydroxy-4-methoxyphenyl)- | - | + | + | + | + | - |
| 26 | 23.983 | 3-(4-Acetoxy-3-methoxyphenyl)-7-methoxy-4-oxo-4H-chromene | + | + | + | + | - | - |
| 27 | 24.716 | Isobenzofuro[5,6-b]benzofuran-8-carboxylic acid, 1,3-dihydro-7,10-dimethoxy-9-methyl-1-oxo-, methyl ester | + | - | - | + | - | - |
| 28 | 27.521 | Phenanthro[3,2-b]furan-7,11-dione, 1,2,3,4,8,9-hexahydro-4,4,8-trimethyl-, (+)- | + | + | - | + | - | - |
| 29 | 28.579 | Oxirane, 2,2'-[(1-methylethylidene)bis(4,1-phenyleneoxymethylene)]bis- | - | - | + | + | - | - |
| 30 | 28.784 | [1,1'-Biphenyl]-3,3'-dicarboxaldehyde, 6,6'-dihydroxy-5,5'-dimethoxy- | - | - | + | + | + | - |
| **Furan** | | |  |  |  |  |  |  |
| 31 | 13.453 | Ethanone, 1-[3-[2-methyl-2-(5-methyl-2-furanyl)propyl]oxiranyl]- | - | - | + | + | + | - |
| **Aldehydes** | | |  |  |  |  |  |  |
| 32 | 12.115 | Dodecan-1-yl acetate | + | - | - | - | - | - |
| 33 | 27.198 | 3-Buten-2-one, 4-(2,6,6-trimethyl-1-cyclohexen-1-yl)- | - | - | + | + | + | - |
| **Esters** | | |  |  |  |  |  |  |
| 34 | 5.135 | Pentanoic acid, ethyl ester | + | + | + | + | + | + |
| 35 | 5.513 | Propanedioic acid, dimethyl ester | + | + | + | + | + | + |
| 36 | 12.806 | 2-Propenoic acid, oxybis(methyl-2,1-ethanediyl) ester | + | - | + | + | + | + |
| 37 | 15.19 | Hexadecanoic acid, methyl ester | + | - | + | + | - | + |
| 38 | 17.79 | Octadecanoic acid, methyl ester | + | - | + | - | + | + |
| **Organic acids** | | |  |  |  |  |  |  |
| 39 | 6.386 | Butanoic acid, 3-hydroxy- | - | - | - | + | - | - |
| 40 | 15.6 | n-Hexadecanoic acid | + | + | + | + | + | + |
| 41 | 18.254 | Octadecanoic acid | + | + | + | + | + | + |
| **Alkanes** | | |  |  |  |  |  |  |
| 42 | 7.465 | Undecane | + | + | + | + | + | + |
| 43 | 9.483 | Hexane, 2,3,4-trimethyl- | - | - | - | + | - | - |
| 44 | 17.456 | Nonadecane | + | - | + | - | + | - |
| 45 | 18.847 | Docosane | + | - | - | - | - | - |

^a^ Note: “RT”, retention time; “min”, minute, “-”, compound was not detected; “+”, compound was detected; “L”, Lignin control; “L+H”, Lignin with the presence of 0.1 mM H_2_O_2_; “S+L”, Secretome with lignin; “S+L+H”, Secretome with the presence of lignin and 0.1 mM H_2_O_2_; “S+L+H+M”, Secretome with the presence of lignin, 0.1 mM H_2_O_2_ and 0.1 mM Mn^2+^; “S”, Secretome control, Lignin concentration was set as 2 g·L^-1^ for all treatments. Structures are shown in the supplementary material, Figure S3.

Table S2. Grouped lignin breakdown products among all treatments ^a^.

|  | **Peak area** | | | |
| --- | --- | --- | --- | --- |
|  | **Increased** | **Decreased** | **Degraded** | **Not changed** |
| **Lignin + H_2_O_2_** | 1, 3, 4, 5, 6, 7, 9, 11, 12, 14, 16, 17, 18, 20, 21, 24, 25, 26, 34, 35, 42 | 40 | 2, 13, 15, 19, 22, 27, 32, 36, 37, 38, 44, 45 | 23, 28 |
| **Lignin+Secretome** | 3, 7, 8, 9, 10, 13, 14, 25, 29, 30, 31, 33 | 1, 2, 5, 11, 15, 16, 20, 22, 23, 24, 26, 34, 35, 36, 37, 38, 40, 41, 42, 44 | 12, 19, 21, 27, 28, 32, 45 | 22 |
| **Lignin+Secretome+H_2_O_2_** | 3, 7, 8, 9, 10, 11, 13, 14, 25, 26, 29, 30, 31, 33, 39, 43 | 1, 2, 5, 15, 16, 20, 22, 24, 27, 28, 34, 35, 36, 37, 40, 41, 42 | 12, 19, 21, 32, 38, 44, 45 | 23 |
| **Lignin+Secretome+H_2_O_2_+Mn^2+^** | 7, 8, 9, 10, 11, 13, 14, 25, 30, 31, 33, 36 | 1, 2, 5, 11, 15, 16, 20, 22, 23, 24, 34, 35, 38, 40, 41, 42, 44 | 3, 12, 19, 21, 26, 27, 28, 32, 37, 45 | - |

^a^ Note: Peak area alterations (increased and decreased) were compared to original lignin control. The degraded group indicated that the compounds were not detected in the treatment but in the original lignin control.

Table S3. Main lignin 2D ^1^H-^13^C Cross-peak assignments in the HSQC Spectra.^1-3^

| **Label** | **δH (ppm)** | **δC (ppm)** | **Assignments** |
| --- | --- | --- | --- |
| -OMe | 3.70 | 55.6 | C-H in methoxyls |
| A_α_ | 4.83 | 71.6 | C_α_-H_α_ in β-O-4' substructures (A) |
| A_β(G)_ | 4.34 | 83.6 | C_β_-H_β_ in β-O-4' substructures linked to G units (A) |
| A_β(S)_ | 4.10 | 85.8 | C_β_-H_β_ in β-O-4' substructures linked to S units (A) |
| A_γ_ | 3.20-3.50 | 59.5-60.1 | C_γ_-H_γ_ in β-O-4' substructures (A) |
| A'_γ_ | 4.20 | 63.5 | C_γ_-H_γ_ in γ-acylated β-O-4' substructures (A) |
| B_α_ | 4.60 | 84.8 | C_α_-H_α_ in resinol (β-β’) substructures (B) |
| B_β_ | 3.05 | 55.1 | C_β_-H_β_ in resinol (β-β’) substructures (B) |
| B_γ_ | 3.87;4.10 | 70.8 | C_γ_-H_γ_ in resinol (β-β’) substructures (B) |
| C_α_ | 5.58 | 87.3 | C_α_-H_α_ in phenylcoumaran (β-5’) substructures (C) |
| C_β_ | 3.49 | 52.3 | C_β_-H_β_ in phenylcoumaran (β-5’) substructures (C) |
| C_γ_ | 3.65 | 61.9 | C_γ_-H_γ_ in phenylcoumaran (β-5’) substructures (C) |
| D_α_ | 4.89 | 81.8 | C_α_-H_α_ in spirodienone (β-1) substructures (D) |
| E_γ_ | 4.07 | 61.6 | C_γ_-H_γ_ in hydroxycinnamyl substructures (E) |
| G_2_ | 6.95 | 111.0 | C_2_-H_2_ in guaiacyl units (G) |
| G_5_ | 6.77 | 115.4 | C_5_-H_5_ in guaiacyl units (G) |
| G_6_ | 6.74 | 118.8 | C_6_-H_6_ in guaiacyl units (G) |
| S_2/6_ | 6.68 | 103.7 | C_2,6_-H_2,6_ in etherified syringyl units (S) |
| S'_2/6_ | 7.30 | 106.3 | C_2,6_-H_2,6_ in oxidized syringyl units (S') |
| H_2/6_ | 7.21 | 128.2 | C_2,6_-H_2,6_ in p-hydroxyphenyl substructures (H) |
| FA_2_ | 7.26 | 111.3 | C_2_-H_2_ in ferulate units (FA) |
| FA_6_ | 7.09 | 121.9 | C_6_-H_6_ in ferulate units (FA) |
| FA_α_ | 7.47 | 143.8 | C_α_-H_α_ in ferulate (FA) |
| FA_β_ | 6.39 | 116.5 | C_β_-H_β_ in ferulic acid (FA) |
| ρCA_2/6_ | 7.44 | 129.9 | C_2,6_-H_2,6_ in ρ-coumarate units (ρCA) |
| ρCA_3/5_ | 6.77 | 115.4 | C_3,5_-H_3,5_ in ρ-coumarate units (ρCA) |
| ρCA_α_ | 7.47 | 143.8 | C_α_-H_α_ in ρ-coumarate (ρCA) |
| ρCA_β_ | 6.23 | 113.7 | C_β_-H_β_ in ρ-coumarate (ρCA) |

**References**

1. J. C. del Río, J. Rencoret, P. Prinsen, Á. T. Martínez, J. Ralph and A. Gutiérrez, *J. Agric. Food Chem*, 2012, **60**, 5922-5935.

2. L. Zhang, Z. Xu, J. R. Cort, T. Vuorinen and B. Yang, *Energy Fuels*, 2020, **34**, 16310-16319.

3. L. Zhang, L. Yan, Z. Wang, D. D. Laskar, M. S. Swita, J. R. Cort and B. Yang, *Biotechnol. Biofuels*, 2015, **8**, 203.
