# Supplementary material for: Understanding of bacterial lignin extracellular degradation mechanisms by Pseudomonas putida KT2440 via secretomic analysis
Source: Biotechnol Biofuels Bioprod. 2022 Oct 31;15:117. doi: 10.1186/s13068-022-02214-x (PMC9620641; doi:10.1186/s13068-022-02214-x)
Supplement: Supplementary file 3 — Additional file 3. A list of abbreviations is included. [file 13068_2022_2214_MOESM3_ESM.docx]

**Abbreviations**

| **Abbreviation** | **Protein/metabolite name** |
| --- | --- |
| Dyp/PP_3248 | Dyp-type peroxidase |
| CopA | multicopper oxidase |
| GST/PP_1644 | glutathione S-transferase |
| gor | glutathione reductase |
| betA | choline dehydrogenase |
| PP_5154 | FAD-binding oxidoreductase |
| aldA/aldB-I | aldehyde dehydrogenase |
| SOD | Superoxide dismutase |
| VAO/PCMH | vanillyl alcohol oxidase/p-cresol methyl hydroxylase |
